# Strategies for Selecting Reliable Reference-Gene Combinations Across 2D and 3D Cell Culture Models

**DOI:** 10.3390/life16101637

**Published:** 2026-09-30

**Authors:** Natalia Grigorova

**Affiliations:** Department of Pharmacology, Animal Physiology, Biochemistry and Chemistry, Faculty of Veterinary Medicine, Trakia University, 6000 Stara Zagora, Bulgaria; nataliya.grigorova@trakia-uni.bg

**Keywords:** RT-qPCR, candidate reference genes, gene expression normalization, 2D and 3D cell culture, culture architecture, cell line-specific effects, AML12 hepatocytes, 3T3-L1 adipocytes

## Abstract

Reliable RT-qPCR normalization in dynamic 2D/3D cell models requires more than identifying genes with low expression variability. In the present study, we evaluated 11 candidate reference genes in AML12 hepatocytes and 3T3-L1 adipocytes cultured under corresponding 2D and 3D conditions to determine whether conventional stability assessment was sufficient for selecting a reliable normalization combination. All candidates passed the initial screening in AML12 cells, whereas *Actb*, *18S*, and *Gapdh* failed one or more predefined stability criteria in 3T3-L1 cells. geNorm pairwise variation indicated that two-gene normalization was sufficient in both models (V2/3 < 0.15). Nevertheless, linear mixed-effects analysis revealed a significant effect of culture condition on 9 of the 11 candidates in AML12 cells and on all 11 candidates in 3T3-L1 cells. Further analyses using PCA, hierarchical clustering, and BestKeeper-based correlation showed that high expression stability and strong inter-reference concordance coexisted with coordinated condition-dependent variation and associations with biologically regulated target genes. Consequently, some of the algorithmically top-ranked pairs did not exhibit the most favorable profile in terms of expression independence, while the choice of normalizer affected both the magnitude and the statistical interpretation of target-gene responses. Based on these findings, we propose a multistep, model-specific strategy that treats expression stability, inter-reference concordance, expression redundancy, condition dependence, and biological independence as distinct characteristics. This approach shifts the focus from selecting the “most stable” genes toward experimental validation of the reference-gene combination itself and of its impact on biological interpretation.

## 1. Introduction

Reverse-transcription quantitative real-time PCR (RT-qPCR) remains one of the most widely used methods for quantitative gene expression analysis because of its high sensitivity, specificity, and relative technical accessibility. The reliability of relative quantification, however, depends not only on RNA quality, reverse-transcription efficiency, and amplification performance, but also critically on the strategy used for data normalization [1]. This issue is not fully resolved even by digital PCR (dPCR). Although dPCR enables direct quantification of target molecules without relying on Cq values or standard curves, pre-analytical sources of variability, including RNA extraction and reverse transcription, remain and still require an appropriate normalization strategy when biological samples are compared [2]. The updated MIQE 2.0 guidelines emphasize that both the selection and number of reference genes should be experimentally justified and that their stability should be demonstrated within the specific biological system and experimental conditions [3].

Accordingly, the traditional classification of a gene as “housekeeping,” implying involvement in fundamental cellular processes and relatively constitutive expression, does not by itself guarantee suitability for RT-qPCR normalization. In this context, the term candidate reference gene is more appropriate. Any potential internal control should initially be considered a candidate whose suitability must be demonstrated within the specific biological system and experimental design.

*Gapdh*, *Actb*, and *18S* rRNA illustrate three biologically distinct sources of potential instability in commonly used internal controls. None of these genes are intrinsically unsuitable. Rather, their performance changes with experimental context. *Gapdh*, a central glycolytic gene, has been validated as stable in several hepatic and developmental models [4,5,6,7], yet it becomes markedly variable during 3T3-L1 adipogenesis, where glucose utilization and energy metabolism are extensively remodeled [8,9,10]. *Actb* shows a similar context dependence. It can remain stable during differentiation of human subcutaneous adipocytes [11], but becomes unreliable in models involving cytoskeletal reorganization, altered cell morphology, hypoxia, or extracellular matrix remodeling [12,13,14,15]. *18S* rRNA presents a different limitation. Its transcriptional regulation and abundance differ fundamentally from those of most mRNA targets [16,17], and, although it has been reported as stable during standard 3T3-L1 differentiation [9], it became one of the least stable candidates in a more complex matrix- and differentiation-dependent model [15]. Together, these contrasting observations show that neither conventional use nor previous successful validation can be automatically extrapolated to other experimental systems. The suitability of a housekeeping gene must therefore be demonstrated for the specific biological and experimental context in which it is used [18].

Experimental validation of candidate reference genes, however, does not fully resolve the problem because the most widely used algorithms assess different and partly complementary aspects of expression stability, and their rankings do not necessarily translate directly into equivalent normalization performance [19]. NormFinder applies model-based variance estimation and considers intra- and inter-group variation to identify the most stable individual gene and an optimal two-gene combination [20]. Although well-suited to detecting systematic group-dependent variation, it is not designed to construct larger functionally independent normalization panels or to assess relationships between candidate and target genes. geNorm evaluates stability by examining the consistency of expression ratios among candidate reference genes and determines the optimal number of genes through sequential pairwise variation [21]. This approach carries the risk that co-regulated genes may appear highly stable relative to one another even when they change in parallel in response to the experimental factor [21,22]. BestKeeper evaluates Cq variability and pairwise correlations and constructs a BestKeeper Index from the most suitable candidates [23]. If high correlation with the BestKeeper Index is used as the sole criterion of stability, particularly when the candidate set contains co-regulated genes, an apparently robust panel may emerge even though its members follow the same biological trend. The original BestKeeper framework also permits evaluation of target genes in relation to the index. Thus, the limitation lies less in the algorithm itself than in reducing its use to a rapid ranking procedure without subsequent assessment of biological independence [23]. The comparative ΔCt approach focuses primarily on consistency among candidate genes; whereas, RefFinder integrates results from several methods but does not eliminate their individual limitations [24]. These tools provide rapid rankings, but such rankings should not be equated with complete validation of biological suitability [14,15,24].

At present, no single algorithm can independently address all practical requirements for a reliable internal control in a complex biological experiment. Low Cq variability alone is insufficient. An appropriate reference gene should show limited expression variability, show no systematic dependence on the major factors of the experimental design, and be as independent as possible from the biological process reflected by the target genes. When more than one internal control is used, the functional and expression dependence among members of the normalization panel should also be considered. Reliable selection cannot be reduced to choosing the “winner” of a single ranking or mechanically comparing several rankings. Instead, it requires a sequential, multistep strategy for candidate exclusion and confirmation that integrates overall variability, factor-dependent effects, expression concordance, and biological independence, followed by functional evaluation of how the selected panel influences normalized target-gene expression. Chervoneva et al. demonstrated that independence among candidate reference genes cannot be assumed and that an optimal normalization set is not necessarily obtained by simply combining the individually highest-ranked genes [22]. Moreover, the choice among formally acceptable reference genes has been shown to alter not only the magnitude of the target gene response but also the detection or loss of statistical significance [25,26].

This issue becomes particularly important in contemporary in vitro models, where differentiation, transitions between 2D and 3D organization, extracellular matrix composition, and changes in cell adhesion and mechanical signaling can simultaneously reshape the cytoskeleton, energy metabolism, ribosome biogenesis, and transcriptional activity. Many traditional reference genes participate directly or indirectly in these processes, making their expression potentially sensitive to changes in culture architecture. This concern is supported by previous 2D/3D studies showing that culture dimensionality can alter the stability of commonly used reference genes [27,28], while recent transcriptome-wide analysis of collagen I-based 3D cultures further demonstrated extensive, cell-specific transcriptional reprogramming accompanied by altered expression of conventional housekeeping genes [29]. Complex multifactorial cell models therefore provide a particularly relevant setting in which to evaluate validation strategies that extend beyond conventional algorithm-based ranking.

In the present study, the expression stability of eleven widely used candidate reference genes was evaluated in parallel in two biologically distinct murine cell systems, AML12 and 3T3-L1, cultured under comparable 2D and 3D conditions. The primary aim was to determine whether a sequential multistep strategy combining conventional stability algorithms with direct assessment of culture-condition effects, multivariate expression patterns, and co-variation with biologically relevant target genes could improve the selection of reliable normalization pairs. Rather than seeking a universal housekeeping gene, the study aimed to establish and experimentally evaluate a practical framework for selecting biologically appropriate internal controls in complex 2D/3D RT-qPCR models.

## 2. Materials and Methods

### 2.1. AML12 Cell Culture and Experimental Conditions

The murine hepatocyte cell line AML12 (Cat. No. 300643, Cytion, Heidelberg, Germany) was propagated in Ham’s F12 medium (Cat. No. 820400a, Cytion, Heidelberg, Germany), supplemented with 10% fetal bovine serum (FBS, Cat. No. F7524 Sigma-Aldrich Chemie GmbH, Merck KGaA, Darmstadt, Germany), 1% insulin–transferrin–selenium (ITS, Cat. No. I3146, 100×Sigma-Aldrich Chemie GmbH, Merck KGaA, Darmstadt, Germany), 1% antibiotic–antimycotic solution containing penicillin, streptomycin, and amphotericin B (Cat. No. A5955, Sigma-Aldrich Chemie GmbH, Merck KGaA, Darmstadt, Germany), and 40 ng/mL dexamethasone (Cat. No. D4902, Sigma-Aldrich Chemie GmbH, Merck KGaA, Darmstadt, Germany). Routine cultures were maintained in T75 flasks at 37 °C in a humidified atmosphere containing 5% CO_2_ and were subcultured at approximately 80% confluence.

For the comparative experiment, AML12 cells were established under four culture configurations: standard tissue-culture-treated plastic (TC), a two-dimensional type I collagen-coated surface (2D Coll), a thick collagen gel substrate (3D Coll), and a cell-containing collagen microsphere configuration (Sph Coll). All experimental cultures were initiated with 2 × 10^4^ cells per well and maintained under the respective conditions for 7 days.

Biological replicates were generated from different cell passages and independent seeding runs performed at different times under otherwise identical experimental conditions, thereby incorporating between-passage and between-run variability. Culture medium was renewed every 48 h. At the end of the 7-day culture period, cellular metabolic activity was assessed using a resazurin-based assay immediately before sample collection for subsequent molecular analyses.

### 2.2. 3T3-L1 Cell Culture and Adipogenic Differentiation

Murine 3T3-L1 preadipocytes (Cat. No. CRL-3242, American Type Culture Collection, Washington, DC, USA) were expanded in high-glucose Dulbecco’s Modified Eagle’s Medium (DMEM-HG; 4.5 g/L glucose, Cat. No. D5796, Sigma-Aldrich Chemie GmbH, Merck KGaA, Darmstadt, Germany) supplemented with 10% FBS and 1% antibiotic–antimycotic mixture containing penicillin G, streptomycin, and amphotericin B. Routine cultures were maintained at 37 °C and 5% CO_2_ in a humidified incubator.

Experimental cultures were established using the same four configurations as for AML12 cells (TC, 2D Coll, 3D Coll, and Sph Coll), with an identical initial cell number of 2 × 10^4^ cells per experimental unit. Since proliferative kinetics differed among the culture architectures, adipogenic induction was synchronized according to cellular growth status rather than to a predefined number of days after seeding. Cell proliferation was followed daily for each configuration, and differentiation was initiated only after the cell number remained unchanged for 24 h, indicating growth arrest. Consequently, individual culture models could enter the differentiation protocol at different chronological times despite having been initiated with the same number of cells.

At this model-specific starting point, adipogenesis was induced with DMEM-HG supplemented with 10 μg/mL insulin, 0.1 mM 3-isobutyl-1-methylxanthine (IBMX, Cat. No. I5879, Sigma-Aldrich Chemie GmbH, Merck KGaA, Darmstadt, Germany), 1 μM dexamethasone (Cat. No. D4902, Sigma-Aldrich Chemie GmbH, Merck KGaA, Darmstadt, Germany), and 0.05 mM indomethacin (Cat. No. I7378, Sigma-Aldrich Chemie GmbH, Merck KGaA, Darmstadt, Germany). After 48 h of induction, the medium was replaced with maintenance medium consisting of the basal culture medium supplemented with 10 μg/mL insulin (Cat. No. I0516, Sigma-Aldrich Chemie GmbH, Merck KGaA, Darmstadt, Germany). Cells were subsequently maintained in this medium for the remainder of the differentiation period.

As for AML12 cells, replicate cultures originated from different passages and independent seeding runs performed on separate occasions, while all experimental procedures were kept unchanged. All 3T3-L1 cultures were evaluated after 7 complete days of differentiation, irrespective of the chronological time required by each architecture to reach the initial growth-arrest criterion. A resazurin-based metabolic assay was performed at the end of day 7 immediately before sample collection.

### 2.3. Establishment of 2D and 3D Collagen-Based Culture Models

For the TC condition, cells were seeded directly onto standard tissue-culture-treated 24-well plates (Cat. No.9224, TPP, Trasadingen, Switzerland). In contrast, all collagen-containing models were prepared in non-treated, flat-bottom 24-well plates (Cat. No.351147, Falcon, Corning Inc., Corning, NY, USA), minimizing adhesion to the underlying plastic and allowing cellular organization to be determined predominantly by the collagen configuration.

For 2D Coll, type I rat-tail collagen (Cat. No. 354236, Corning Inc., Corning, NY, USA) was diluted to 5 μg/mL in 0.02 N acetic acid (Cat. No. 34256, Honeywell Specialty Chemicals Seelze GmbH, Seelze, Germany). Each well received 150 μL of the collagen solution and was incubated at 37 °C for 30 min. Residual coating solution was then removed, the wells were rinsed with PBS (Cat. No. 59331C, DPBS without Ca^2+^ and Mg^2+^, Corning Inc., Corning, NY, USA), and 2 × 10^4^ cells were seeded onto the collagen-coated surface.

The 3D Coll configuration was generated using a substantially thicker collagen layer. Type I rat-tail collagen was adjusted to a final concentration of 1.5 mg/mL in PBS and neutralized with 1 N NaOH (Cat. No. S2770; Sigma-Aldrich Chemie GmbH, Merck KGaA, Darmstadt, Germany) before use. A 200 μL aliquot was dispensed into each well and incubated for 40 min to permit complete gel formation. Subsequently, 2 × 10^4^ cells were placed directly onto the polymerized collagen matrix. The cultures were maintained without medium for an additional 30 min to allow the cells to settle onto the gel surface, after which 600 μL of the appropriate AML12 or 3T3-L1 culture medium was carefully added.

For the Sph Coll model, the neutralized type I collagen preparation was likewise adjusted to 1.5 mg/mL. A cell suspension prepared in DMEM was incorporated into the collagen mixture so that each 20 μL aliquot contained 2 × 10^4^ cells. One cell-containing collagen microdroplet was deposited centrally in each pre-incubated non-treated well. The constructs were allowed to polymerize for 30 min, generating one discrete collagen microsphere per well, after which 600 μL of the respective culture medium was gently added to surround the construct.

Culture media were renewed every 48 h in all four configurations. Apart from the cell line-specific basal media and the adipogenic differentiation protocol applied to 3T3-L1 cells, the procedures used to generate TC, 2D Coll, 3D Coll, and Sph Coll were identical for AML12 and 3T3-L1 cultures.

At the end of the 7-day experimental period, the morphology and spatial organization of both AML12 and 3T3-L1 cultures were documented in their native state using an inverted Leica DMi1 microscope equipped with a 5-megapixel digital camera (Leica Microsystems, Wetzlar, Germany). Representative images were obtained for all four culture configurations (TC, 2D Coll, 3D Coll, and Sph Coll).

### 2.4. Resazurin-Based Assessment of Cellular Metabolic Activity

Cellular metabolic activity was evaluated at the end of the 7-day experimental period using a resazurin reduction assay (Cat. No. B21187.06, Resazurin sodium salt, Thermo Fisher Scientific, Waltham, MA, USA). The assay was performed for both AML12 and 3T3-L1 cultures immediately before sample collection. Resazurin reagent was added to the culture medium at a final concentration of 1% (*v*/*v*), and the cells were incubated for 2 h at 37 °C in a humidified atmosphere containing 5% CO_2_.

Following incubation, 150 µL of culture supernatant from each well was transferred to a 96-well plate. Resorufin fluorescence was quantified using a Synergy™ LX Multi-Mode Microplate Reader (BioTek Instruments, Inc., Winooski, VT, USA) at an excitationwavelength of 560 nm and an emission wavelength of 590 nm. Fluorescence intensity was used as an indirect measure of the metabolic reduction capacity of the viable cell population under the respective culture conditions.

### 2.5. RNA Isolation, Quality Assessment, and cDNA Synthesis

Total RNA was isolated from AML12 and 3T3-L1 cultures at the end of the 7-day experimental period using the RNeasy Plus Mini Kit (Cat. No. 74134, QIAGEN GmbH, Hilden, Germany), following the manufacturer’s protocol. The same RNA isolation procedure was applied to both cell lines and across all culture configurations. Genomic DNA was removed during RNA purification using the gDNA Eliminator spin columns included in the kit prior to RNA binding and subsequent column purification.

RNA concentration and purity were evaluated spectrophotometrically using a Take3 Microvolume Plate with a Synergy™ LX Multi-Mode Microplate Reader (BioTek Instruments, Inc., Winooski, VT, USA). RNA purity was assessed from the A260/A280 absorbance ratio, which ranged from 1.90 to 2.118 across the analyzed samples. Following quantification, RNA input was standardized to 500 ng per reverse-transcription reaction.

First-strand cDNA was synthesized using the RevertAid First Strand cDNA Synthesis Kit (Cat. No. K1612, Thermo Fisher Scientific, Waltham, MA, USA) with oligo(dT)18 primers. To minimize the technical variation associated with reverse-transcription batch effects, all samples from the same cell line were reverse-transcribed within the same experimental run under identical conditions. The resulting cDNA was stored at −20 °C for less than one month and diluted 1:50 with nuclease-free water before RT-qPCR analysis.

### 2.6. RT-qPCR Analysis, Primer Design and Validation

RT-qPCR was performed using KAPA SYBR Fast qPCR Master Mix (Kapa Biosystems, Merck KGaA, Darmstadt, Germany) on a PikoReal Real-Time PCR System (Thermo Fisher Scientific, Waltham, MA, USA). Reactions were prepared in a final volume of 10 µL and analyzed in technical duplicate. The same reaction composition and amplification protocol were applied to both AML12 and 3T3-L1 samples. Quantification cycle values are reported throughout the study as Cq.

Primer pairs were designed against the corresponding Mus musculus transcript sequences using NCBI resources and Primer3Plus version 3.2.0. Candidate sequences were screened in silico for target specificity and expected amplicon characteristics using NCBI Primer-BLAST (National Center for Biotechnology Information, Bethesda, MD, USA; web-based tool, https://www.ncbi.nlm.nih.gov/tools/primer-blast/, accessed on 10 August 2026) and complementary primer-analysis tools. The final primer sequences, accession numbers, and expected amplicon sizes are provided in Table 1.

Before analysis of the experimental samples, each assay was empirically validated using pooled cDNA prepared separately from AML12 and 3T3-L1 samples. Amplification specificity was evaluated by agarose gel electrophoresis, confirming amplification products of the expected size, and by post-amplification melting-curve analysis, which showed a single predominant melting peak for each primer pair. No-template controls (NTCs), in which the cDNA template was replaced with nuclease-free water, were included in the RT-qPCR runs to monitor reagent contamination and nonspecific amplification.

Amplification was performed using the following cycling program: initial denaturation at 95 °C for 3 min, followed by 40 cycles of denaturation at 95 °C for 3 s and annealing/extension at 60 °C for 30 s, with fluorescence acquisition at the end of each annealing/extension step. Amplification was followed by a 30 s hold at 60 °C and melting-curve analysis from 60 to 95 °C, with 0.2 °C temperature increments and a 1 s hold at each step. Fluorescence acquisition and Cq determination were performed using PikoReal Software 2.2 (Thermo Fisher Scientific, Waltham, MA, USA).

Gene-specific amplification efficiencies were established independently for AML12 and 3T3-L1 assays using standard curves generated from serial dilutions of pooled cDNA from the corresponding cell line. Amplification efficiencies for all assays in AML12 and 3T3-L1 cells are reported in Supplementary Table S1.

Eleven genes were evaluated as candidate reference genes in both cell lines: 18S ribosomal RNA (*18S*), β-actin (*Actb*), beta-2 microglobulin (*B2m*), glyceraldehyde-3-phosphate dehydrogenase (*Gapdh*), hydroxymethylbilane synthase (*Hmbs*), hypoxanthine guanine phosphoribosyltransferase (*Hprt*), non-POU domain-containing octamer-binding protein (*Nono*), peptidylprolyl isomerase A (*Ppia*), ribosomal protein lateral stalk subunit P0 (*Rplp0*), TATA-box binding protein (*Tbp*), and tyrosine 3-monooxygenase/tryptophan 5-monooxygenase activation protein zeta (*Ywhaz*). Target-gene expression was assessed using albumin (*Alb*), hepatocyte nuclear factor 4 alpha (*Hnf4a*), integrin subunit beta 1 (*Itgb1*), and cellular communication network factor 1 (*Ccn1*) in AML12 cells, and fatty acid-binding protein 4 (*Fabp4*), fatty acid synthase (*Fasn*), *Itgb1*, and *Ccn1* in 3T3-L1 cells.

### 2.7. Reference-Gene Stability Evaluation

Expression stability of the 11 candidate reference genes was evaluated separately in AML12 and 3T3-L1 cells using NormFinder, geNorm, and BestKeeper as the primary screening approaches. NormFinder was applied using the original Microsoft Excel add-in (Molecular Diagnostic Laboratory, Aarhus University Hospital, Denmark) [20] to rank individual genes and identify the optimal two-gene combination. Lower stability values indicate greater expression stability, and a value < 0.5 was used as the predefined screening criterion. geNorm analysis was performed in R version 4.6.1 (R Foundation for Statistical Computing, Vienna, Austria) using the NormqPCR package (version 1.58.0; Bioconductor 3.23) [30]. Efficiency-corrected relative quantities were analyzed with selectHKs using the geNorm method and log = FALSE. Candidate stability was evaluated from the M value, with M < 1.0 used as a stringent screening threshold [31]. The conventional M < 1.5 criterion was retained for reference [21]. Pairwise variation (Vn/Vn + 1) was additionally calculated to estimate the number of reference genes required for normalization, using V < 0.15 as the conventional cut-off [21].

BestKeeper was used for both descriptive stability screening and correlation/regression analysis [23]. Cq variability of each candidate reference gene was initially assessed using the standard deviation (SD) and coefficient of variation (CV), with SD < 1 Cq applied as the classical stability criterion and CV < 5% as an additional stringent descriptive threshold. Candidates fulfilling the predefined screening criteria were subsequently subjected to BestKeeper correlation analysis. Their concordance with the BestKeeper Index was evaluated using the pair-wise correlation/regression procedure implemented in the software. Preselected reference-gene combinations were then assessed by constructing pair-specific BestKeeper indices and examining the correlation of each component gene with the corresponding index as a measure of within-pair concordance. In a second correlation/regression analysis, each pair-specific BestKeeper Index was evaluated against the respective target genes to assess potential co-variation with biologically regulated expression. Direct reference-gene–target correlations were additionally examined, where informative.

The primary stability assessment was supplemented by the comparative ΔCq method of Silver et al. [32] and RefFinder [33] (https://www.ciidirsinaloa.com.mx/RefFinder-master/; accessed on 10 August 2026). RefFinder integrates rankings generated by geNorm, NormFinder, BestKeeper, and the comparative ΔCq method. Because both supplementary analyses were performed directly on Cq values and did not explicitly incorporate gene-specific amplification efficiencies, they were used as complementary robustness checks rather than as independent exclusion criteria.

### 2.8. Hierarchical Clustering and Principal Component Analysis

The structure of reference-gene expression variability was further examined separately in AML12 and 3T3-L1 cells using hierarchical clustering and principal component analysis (PCA) as complementary multivariate approaches. For hierarchical clustering, Cq values were standardized to z-scores within each cell model. Clustering was performed in IBM SPSS Statistics, version 26 (IBM Corp., Armonk, NY, USA) using Ward’s linkage method and squared Euclidean distance. Candidate reference genes were clustered according to their standardized expression profiles across the 20 samples, and dendrograms were used to identify groups of genes displaying similar patterns of variation across culture conditions.

For PCA, the original Cq values were entered into GraphPad Prism, version 11.1.0 (GraphPad Software, LLC, Boston, MA, USA) and analyzed after standardization within the PCA procedure. Principal components with eigenvalues > 1 were retained according to the Kaiser criterion. Sample scores were examined to characterize the major condition-associated sources of variation; whereas, gene loadings were used to assess the direction and relative contribution of individual reference genes to the retained components.

### 2.9. Normalization Strategy and Relative Expression Analysis

Final validation of the selected reference-gene combinations was performed by examining their effect on target-gene expression. Target-gene expression was calculated using an efficiency-corrected relative quantification approach and normalized to the geometric mean of the relative quantities of the selected reference-gene pair [21,34]. For single-gene normalization, the corresponding reference-gene relative quantity was used. Expression values were subsequently calibrated to the mean of the TC group and expressed as fold change relative to TC. For graphical presentation and statistical analysis, fold-change values were log_2_-transformed, so that a value of 0 represented expression equivalent to the TC calibrator.

Normalization scenarios included algorithm-derived and integratively selected reference-gene pairs, selected single-gene normalizers, and conventional reference genes (Actb and Gapdh) for comparison. Target-gene relative quantities were also examined after efficiency correction and calibration to TC, but without reference-gene normalization. These target-only profiles served solely as a conditional descriptive reference for evaluating normalization-dependent changes and were not interpreted as a measure of true transcript abundance.

### 2.10. Statistical Analysis

Statistical analyses were performed using IBM SPSS Statistics version 26 (IBM Corp., Armonk, NY, USA) and GraphPad Prism version 11.1.0 (224) (GraphPad Software, LLC, Boston, MA, USA).

To assess condition-dependent variation in candidate reference-gene expression, Cq values were analyzed separately for each gene and cell line using linear mixed-effects models in SPSS. Culture condition was included as a fixed effect and as the repeated factor, with biological replicate specified as the subject to account for the corresponding measurements obtained across the four culture conditions. A compound-symmetry covariance structure was used to model within-replicate correlations. Models were estimated using restricted maximum likelihood (REML), with denominator degrees of freedom calculated using the Satterthwaite approximation. The overall effect of culture condition was evaluated using Type III tests of fixed effects. Estimated marginal means ± SE were reported, and pairwise comparisons among culture conditions were adjusted using the Bonferroni procedure.

All subsequent statistical analyses were performed in GraphPad Prism. Pearson correlation coefficients (r) with corresponding two-sided *p*-values were used for the BestKeeper-related and direct reference-gene–target correlation analyses described above. Target-gene expression was analyzed separately for each target and cell line. Comparisons among culture conditions were performed on log_2_(fold-change) values using ordinary one-way ANOVA followed by Dunnett’s multiple-comparisons test against the TC group. The same procedure was applied to the efficiency-corrected, TC-calibrated target-only profiles used for comparison of normalization-dependent expression patterns.

Resazurin fluorescence data were analyzed separately for each cell line using the Kruskal–Wallis test followed by Dunn’s multiple-comparisons test. Unless otherwise specified, data are presented as mean ± SD from five biological replicates. Statistical significance was accepted at *p* < 0.05.

## 3. Results

### 3.1. Morphological Characteristics and Metabolic Activity of AML12 and 3T3-L1 Cells

**Figure 1:**
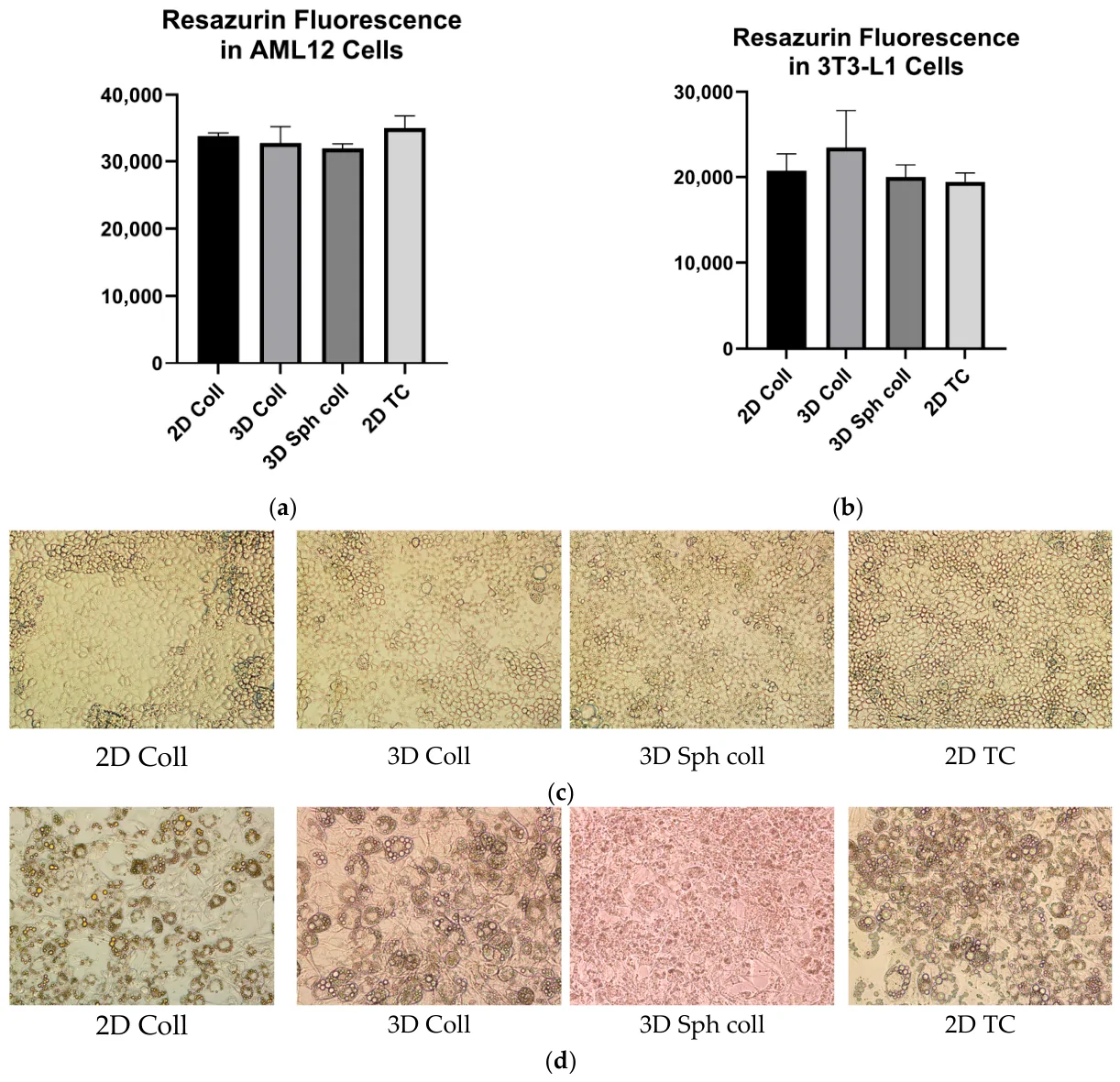
Morphology and metabolic activity of AML12 and 3T3-L1 cells under different culture conditions. Metabolic activity was evaluated by the resazurin reduction assay in (**a**) AML12 and (**b**) 3T3-L1 cells. Cell morphology under the different culture conditions was visualized by bright-field microscopy, with representative images shown for (**c**) AML12 and (**d**) 3T3-L1 cells. Images were acquired at 20× magnification. Resazurin data were analyzed using the Kruskal–Wallis test. Statistical significance was accepted at *p* < 0.05. Abbreviations: 2D TC, two-dimensional culture on standard tissue-culture plastic; 2D Coll, two-dimensional culture on collagen; 3D Coll, three-dimensional collagen culture; Sph Coll, collagen spheroid culture.

No significant differences in resazurin fluorescence were detected among the culture conditions in either AML12 or 3T3-L1 cells (Figure 1a,b). Bright-field microscopy confirmed preserved cellular morphology compatible with the respective 2D and 3D culture formats, without indications of compromised cell condition (Figure 1c,d). Together, these findings support that the subsequent differences in gene-expression profiles reflected culture-dependent cellular responses rather than reduced metabolic activity or impaired cell integrity.

### 3.2. Basal Expression Levels and Variability of Candidate Reference and Target Genes

**Figure 2:**
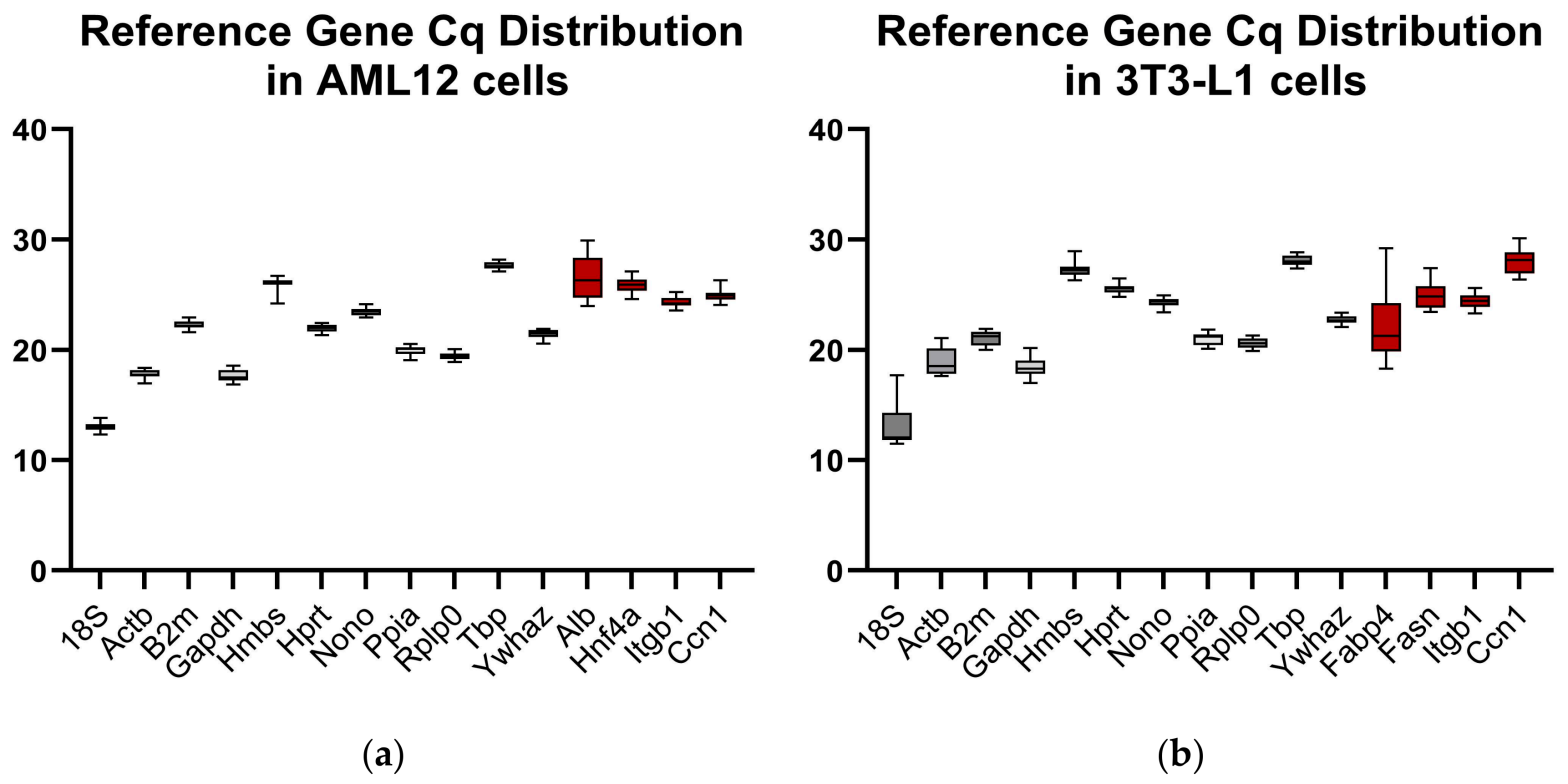
Distribution of Cq values for candidate reference and target genes in AML12 (**a**) and 3T3-L1 (**b**) cells cultured under the respective 2D and 3D experimental conditions. Each condition included five biological replicates (n = 5), for a total of 20 Cq values per gene across the four culture conditions. Box plots indicate the median and interquartile range, with whiskers extending from the minimum to the maximum values. Candidate reference genes are shown in gray and target genes in red.

Candidate reference genes showed broadly comparable expression levels in AML12 and 3T3-L1 cells, with mean Cq values ranging from 13.04 to 27.65 and 12.91 to 28.12, respectively (Figure 2). Individual Cq values for all candidate reference and target genes are provided in Supplementary Tables S2 and S3. For 10 of the 11 genes, the difference in mean Cq between cell lines did not exceed 1.28 cycles. *Hprt* was the only marked exception, showing a 3.58-cycle higher mean Cq in 3T3-L1 than in AML12 cells (25.53 vs. 21.95). In both cell lines, *18S* exhibited the highest expression level, whereas *Tbp* had the lowest.

Notably, the Cq variability was generally higher in 3T3-L1 cells (Figure 2b), particularly for *18S* and *Actb*, whose SD values were approximately 5.0- and 3.3-fold higher than in AML12 cells, followed by *B2m* and *Gapdh* (1.7- and 1.5-fold). The variability of *18S* (SD = 1.85) and *Actb* (SD = 1.27) exceeded that of three of the four target genes in 3T3-L1 cells, with only *Fabp4* showing greater dispersion (SD = 3.25).

### 3.3. Classical Stability Algorithms Identify Context-Dependent Reference-Gene Rankings

**Figure 3:**
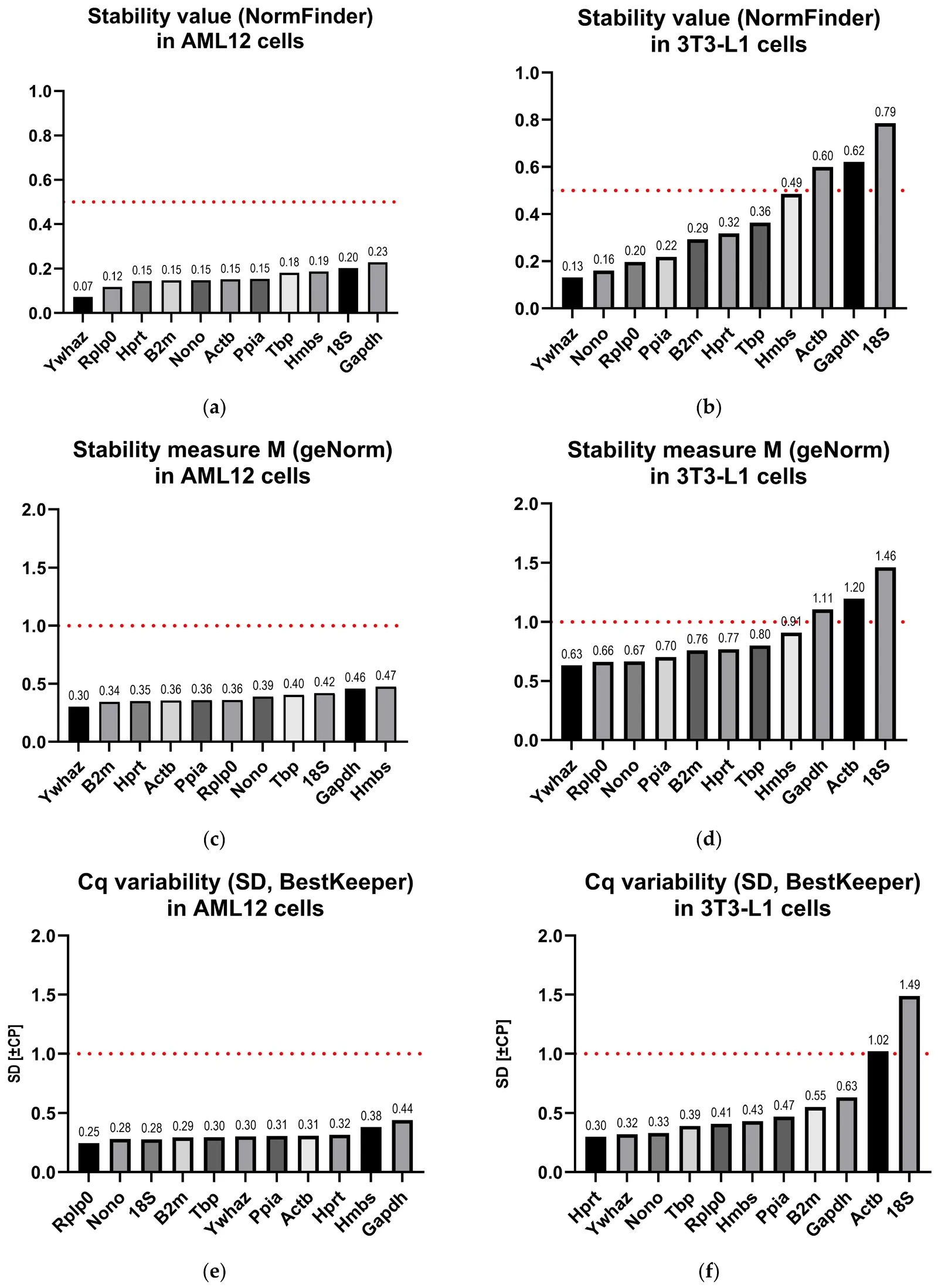

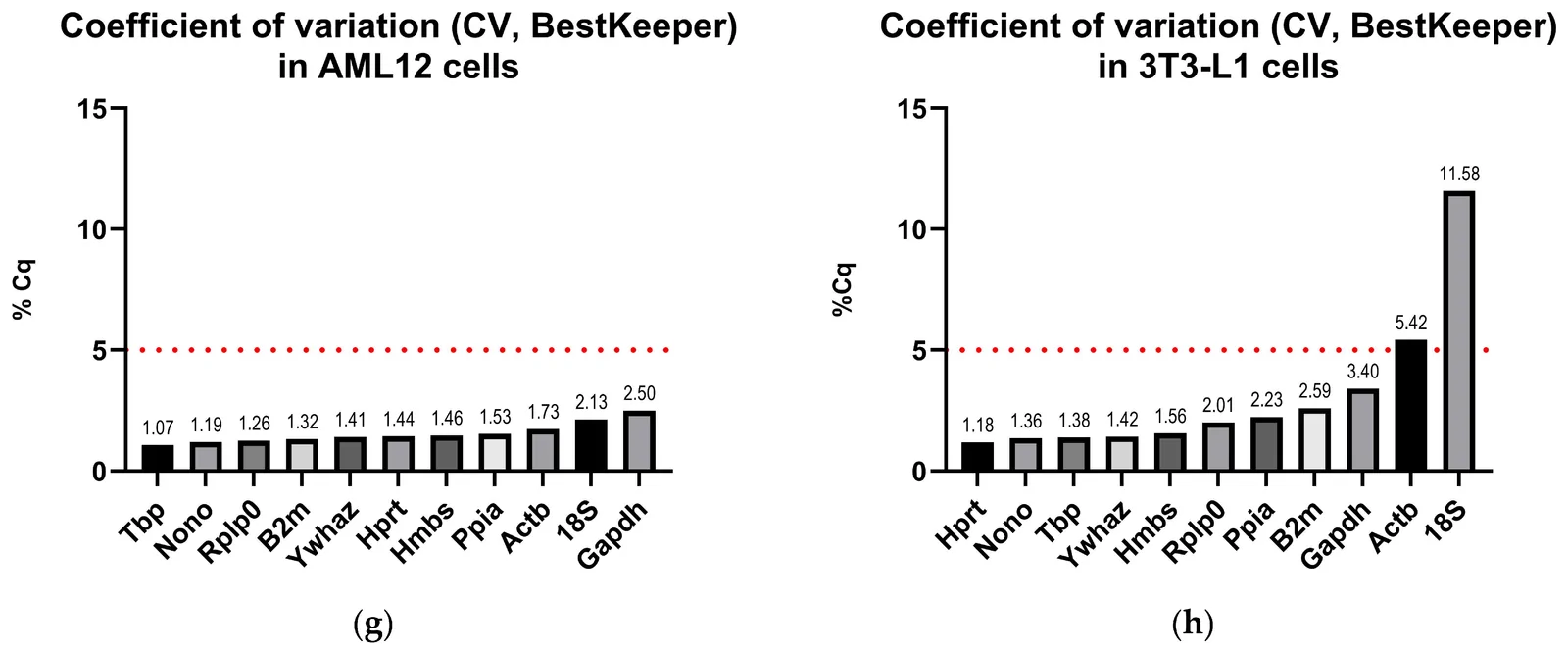
Stability assessment of candidate reference genes in AML12 and 3T3-L1 cells using complementary reference-gene validation approaches. NormFinder stability values in (**a**) AML12 and (**b**) 3T3-L1 cells; geNorm stability measure M in (**c**) AML12 and (**d**) 3T3-L1 cells; BestKeeper standard deviation (SD) of Cq values in (**e**) AML12 and (**f**) 3T3-L1 cells; and BestKeeper coefficient of variation (CV) in (**g**) AML12 and (**h**) 3T3-L1 cells. Red dashed lines indicate the applied stability thresholds: NormFinder stability value = 0.5—a literature-based screening criterion [35], the stringent geNorm M = 1.0 threshold [34], BestKeeper SD = 1 Cq [23], and CV = 5% as an additional stringent descriptive criterion. The conventional geNorm exclusion threshold is M = 1.5 [21]. Lower NormFinder and geNorm M values indicate greater expression stability.

All candidate reference genes in AML12 cells fulfilled the applied stability criteria across all four approaches (Figure 3), including a NormFinder stability value < 0.5 (Figure 3a), geNorm M < 1.0, with all M values in AML12 cells remaining below 0.5 (Figure 3c), BestKeeper SD < 1 Cq (Figure 3e), and CV < 5% (Figure 3g). In contrast, three candidates in 3T3-L1 cells failed at least one criterion. *Actb* and *18S* exceeded the applied limits across NormFinder, geNorm, and BestKeeper, with NormFinder stability values of 0.60 and 0.79 (Figure 3b), geNorm M values of 1.20 and 1.46 (Figure 3d), BestKeeper SD values of 1.02 and 1.49 Cq (Figure 3f), and CV values of 5.42% and 11.58% (Figure 3h), respectively. *Gapdh* also exceeded the NormFinder screening threshold (0.62) and the stringent geNorm threshold (M = 1.11), although its BestKeeper SD and CV remained within the applied limits. *Hmbs* remained just below the NormFinder screening threshold (0.49) and was therefore retained as a borderline candidate for further evaluation. Accordingly, *Actb*, *18S*, and *Gapdh* were excluded from subsequent normalization-panel selection in 3T3-L1 cells.

Among the highest-ranked candidates, NormFinder identified *Ywhaz* as the most stable individual gene in both AML12 and 3T3-L1 cells (Figure 3a,b). geNorm likewise placed *Ywhaz* among the most stable genes, together with *B2m* in AML12 and *Rplp0* in 3T3-L1 cells (Figure 3c,d). BestKeeper yielded partly different rankings: *Rplp0* had the lowest SD in AML12 and *Hprt* in 3T3-L1 cells (Figure 3e,f), whereas CV ranked *Tbp* and *Hprt* first in AML12 and 3T3-L1 cells, respectively (Figure 3g,h).

The primary stability pattern was further examined using RefFinder and the comparative ΔCq method of Silver et al. [32] as supplementary robustness analyses (Supplementary Figures S1 and S2). RefFinder broadly reproduced the rankings obtained with the original stability tools, although its results were not used as an independent exclusion criterion because the platform analyzes raw Cq values without incorporating gene-specific amplification efficiencies, which may lead to differences from the original geNorm and NormFinder implementations [24]. Pairwise ΔCq analysis likewise supported the primary screening outcome, identifying *18S*, *Actb*, and *Gapdh* among the least stable candidates in 3T3-L1 cells. Thus, both supplementary approaches confirmed the reduced stability of the same genes identified by the primary analyses without altering the candidate set retained for subsequent evaluation.

### 3.4. BestKeeper Correlation Analysis Reveals Coordinated Expression Among Candidate Reference Genes

Following the multi-method stability screening, candidate reference genes that met the stability criteria across all applied analyses were subjected to BestKeeper correlation analysis. In AML12 cells, the BestKeeper Index was constructed from ten candidates (given the software limit of ten genes). *Gapdh* was omitted as it showed the least favorable overall stability profile among the other candidates. All included reference genes correlated significantly with the BestKeeper Index (r = 0.550–0.967, *p* ≤ 0.012; Table 2). Importantly, all four AML12 target-genes also correlated significantly with the BestKeeper Index, with r values ranging from 0.466 for *Ccn1* to 0.792 for *Hnf4a,* suggesting possible co-variation between target gene expressions and the reference-gene index.

In 3T3-L1 cells, the initial BestKeeper Index was constructed from the eight candidates remaining after exclusion of *Actb*, *18S*, and *Gapdh*. Five genes (*B2m*, *Nono*, *Ppia*, *Rplp0*, and *Ywhaz*) correlated significantly with the index, whereas *Hmbs*, *Hprt*, and *Tbp* did not (Table 3).

After refinement to these five concordant genes (Table 4), all retained candidates showed strong correlations with the recalculated index (r = 0.889–0.962, all *p* = 0.001).

However, the revised BestKeeper Index showed stronger correlations with the target genes. *Fabp4* and *Fasn* became significantly negatively correlated with the revised index, while the positive correlations of *Itgb1* and *Ccn1* increased to r = 0.810 and 0.818, respectively. These findings indicated that strong concordance with the BestKeeper Index did not necessarily imply independence from target-gene expression, prompting further assessment of the candidate reference-gene pairs.

### 3.5. Direct Reference-Gene–Target-Gene Correlations Reveal Candidate-Specific Relationships

**Figure 4:**
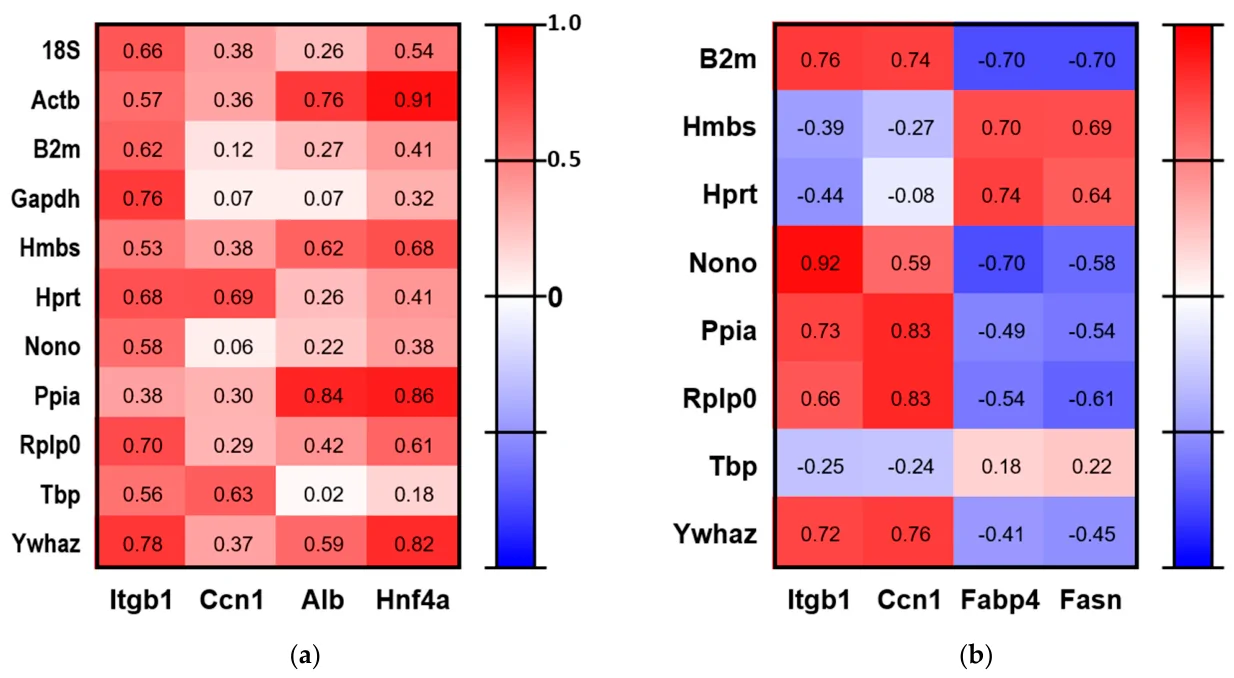
Pearson correlations between candidate reference genes and target genes in AML12 and 3T3-L1 cells. Heatmaps show Pearson correlation coefficients (r) between individual candidate reference genes and the four target genes in (**a**) AML12 and (**b**) 3T3-L1 cells. All 11 candidate reference genes were included for AML12, whereas the 3T3-L1 analysis included the eight candidates retained after the initial stability screening. Correlation coefficients range from −1 to +1, with positive and negative values indicating changes in Cq values in the same and opposite directions, respectively. The corresponding r value is displayed within each heatmap cell.

Following the initial stability screening and BestKeeper-based assessment of inter-reference concordance, direct Pearson correlations were used to examine whether individual candidate reference genes also remained weakly associated with biologically regulated target-gene expression (Figure 4). In AML12 cells, all 11 candidates were included in the analysis because all passed the initial stability screening (Figure 4a). In 3T3-L1 cells, the eight candidates retained after the initial multi-method screening were analyzed, whereas *18S*, *Actb*, and *Gapdh* were not included in this subsequent step because of their lower stability identified in the preceding analyses (Figure 4b).

In AML12 cells, the strength of correlations between individual candidate reference genes and target genes varied considerably. Several candidates showed strong correlations (r ≥ 0.70) with at least one target gene. The most pronounced associations were observed for *Actb* with *Alb* (r = 0.76) and *Hnf4a* (r = 0.91), for *Ppia* with *Alb* (r = 0.84) and *Hnf4a* (r = 0.86), and for *Ywhaz* with *Itgb1* (r = 0.78) and *Hnf4a* (r = 0.82). *Gapdh* also showed a strong correlation with Itgb1 (r = 0.76), whereas *Rplp0* reached r = 0.70 with the same target. In contrast, *18S*, *B2m*, *Hmbs*, *Hprt*, *Nono*, and *Tbp* did not show a strong correlation (r ≥ 0.70) with any target gene. Among these, *Nono* exhibited the most limited overall co-variation (r = 0.06–0.58).

In 3T3-L1 cells, more pronounced and often oppositely directed associations were observed between candidate reference genes and target genes. *B2m* showed positive correlations with *Itgb1* and *Ccn1* (r = 0.76 and 0.74, respectively), but negative correlations with *Fabp4* and *Fasn* (r = −0.70 and −0.70, respectively). A similar pattern was observed for *Nono*, which showed the strongest positive association with *Itgb1* (r = 0.92), together with negative correlations with *Fabp4* (r = −0.70) and *Fasn* (r = −0.58). *Ppia* and *Rplp0* also showed strong positive associations with *Ccn1* (r = 0.83 for both), together with moderate-to-strong negative correlations with the adipogenic targets. In contrast, *Tbp* showed consistently weak correlations with all four target genes (r ranging from −0.25 to 0.22).

Thus, associations of substantial magnitude with target-gene expression were also observed among candidates that showed high stability in the classical algorithms, while both the strength and direction of these relationships differed markedly among individual reference genes and between the two cell lines.

### 3.6. Culture-Condition Dependence of Candidate Reference-Gene Expression

The results of the LMM analysis are presented in Table 5. In AML12 cells, the LMM revealed a significant effect of culture condition for 9 of the 11 candidate reference genes. Only *18S* (F = 0.427, *p* = 0.738) and *Nono* (F = 1.822, *p* = 0.197) showed no significant overall effect. The highest F values were observed for *Gapdh* (F = 34.346, *p* < 0.001) and *Hprt* (F = 34.032, *p* < 0.001).

In 3T3-L1 cells, culture condition had a significant effect on all 11 candidate reference genes, with the strongest effects observed for *Actb* (F = 147.572, *p* < 0.001) and *18S* (F = 59.015, *p* < 0.001). Detailed estimated marginal means and Bonferroni-adjusted pairwise comparisons are presented in Table 6.

### 3.7. Determination of the Optimal Number and Combination of Reference Genes

**Figure 5:**
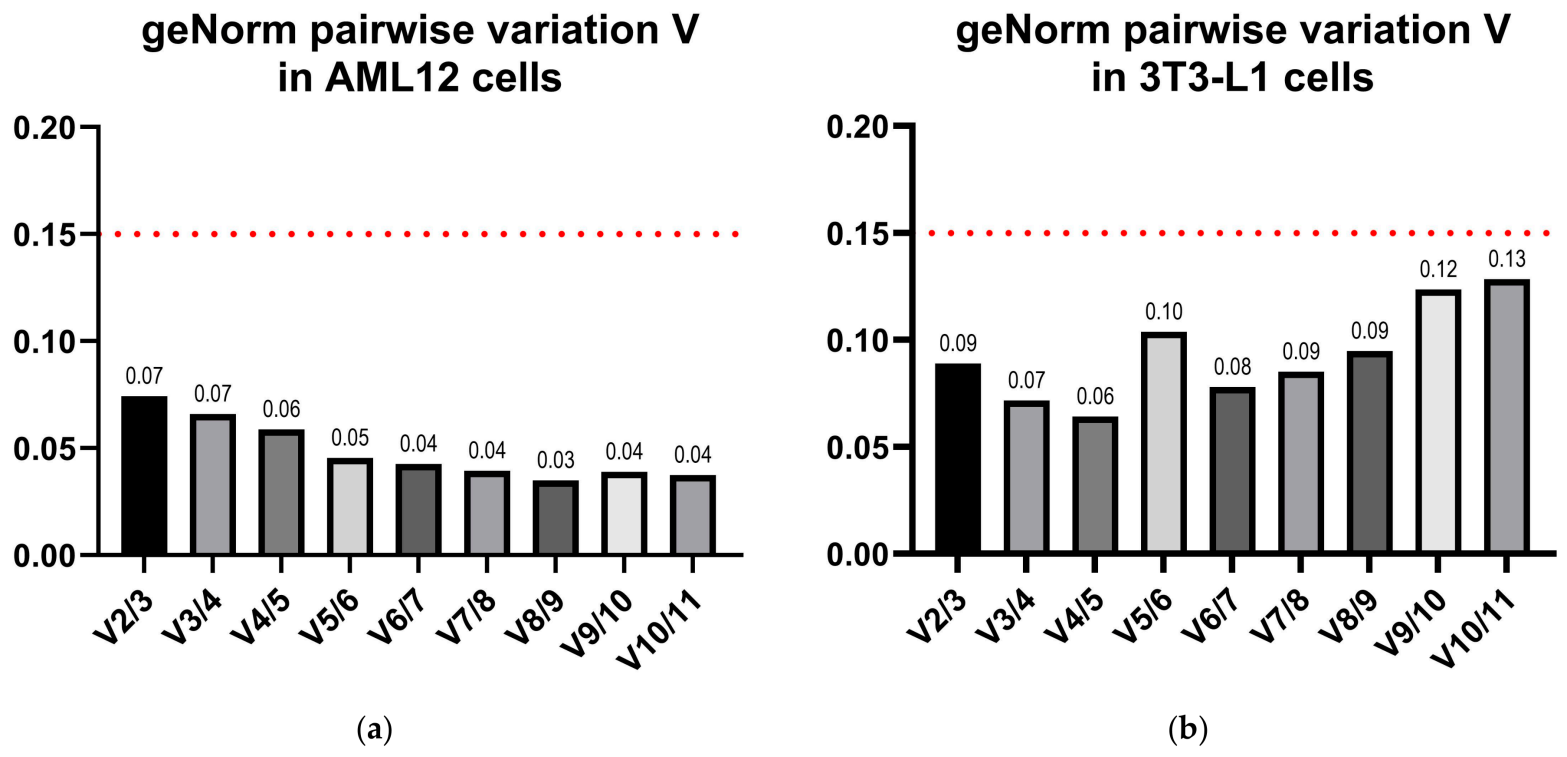
geNorm pairwise variation (Vn/Vn+1) analysis for determination of the optimal number of reference genes in AML12 (**a**) and 3T3-L1 (**b**) cells. The red dotted line indicates the recommended cut-off of V = 0.15 [21]. Values below this threshold indicate that inclusion of an additional reference gene is not required.

geNorm pairwise variation analysis indicated that two reference genes were sufficient for normalization in both cell models (Figure 5). The initial V2/3 values were 0.07 in AML12 and 0.09 in 3T3-L1 cells, both below the recommended 0.15 threshold, and all subsequent Vn/Vn + 1 values remained below this limit.

As geNorm pairwise variation analysis indicated that two reference genes were sufficient for normalization in both cell models, the highest-ranked two-gene sets identified by the different stability approaches were subsequently compared (Table 7).

Overall, the different stability approaches showed only partial agreement in the selection of the highest-ranked two-gene sets. Although *Ywhaz* was repeatedly represented among the top combinations, no single reference-gene pair was consistently identified as optimal across all methods and both cell lines. Thus, the stability rankings alone did not provide a clear basis for selecting a final normalization pair.

### 3.8. BestKeeper-Based Assessment of Reference-Gene Panel Independence from Target-Gene Expression

The reference-gene pairs selected by the preceding stability approaches were therefore evaluated individually for their association with target-gene expression. In AML12 cells, all tested two-gene combinations showed significant correlations of both constituent genes with their respective BestKeeper Index, confirming their contribution to the composite index (Table 8).

However, high ranking by the conventional stability algorithms did not consistently correspond to target-gene independence. *Ywhaz*, identified as the best individual candidate by NormFinder and among the top-ranked genes by geNorm, was significantly associated with *Alb*, *Hnf4a*, and *Itgb1*. Similarly, the NormFinder/geNorm-supported *Ywhaz* + *B2m* pair retained significant associations with *Hnf4a* and *Itgb1*. The *Rplp0* + *Ywhaz* combination, also supported by the supplementary RefFinder and comparative ΔCq analyses, showed a similar pattern of target-gene co-variation. The BestKeeper SD-derived pairs likewise retained significant associations with *Hnf4a* and *Itgb1*. In contrast, *Tbp* + *Nono*, selected from the lowest BestKeeper CV values, showed the most favorable target-gene independence profile, with its composite index unrelated to *Alb*, *Hnf4a*, or *Ccn1* and significantly associated only with *Itgb1*.

In 3T3-L1 cells, the reference-gene pairs selected by the different stability approaches differed markedly in their independence from target-gene expression (Table 9). All constituent genes correlated significantly with their respective BestKeeper Index, with the strongest internal concordance observed for the NormFinder- and geNorm-derived pairs (r = 0.947–0.987). However, these indices were also significantly associated with all four target genes (r = 0.490–0.865), whereas the BestKeeper-derived pairs showed fewer significant target-gene associations.

Because Tbp fulfilled the conventional stability criteria and showed only weak, non-significant direct correlations with all four target genes, we next tested whether its inclusion in the pre-established 3T3-L1 reference-gene pairs could reduce co-variation with target-gene expression. For each pair presented in Table 9, a three-gene BestKeeper Index was recalculated after addition of Tbp, and its association with the same target-gene panel was assessed (Table 10).

The effect of *Tbp* addition was strongly pair dependent. The two *Hprt*-containing combinations, *Hprt* + *Ywhaz* + *Tbp* and *Hprt* + *Nono* + *Tbp*, showed the most favorable target-association profiles, with weak and non-significant correlations with all four target genes. In contrast, significant positive associations with *Itgb1* and *Ccn1* persisted after addition of *Tbp* to the *Nono* + *Ywhaz* and *Ywhaz* + *Rplp0* pairs. Thus, the addition of a third reference gene did not uniformly improve target-gene independence but depended on the composition of the underlying reference-gene pair.

### 3.9. Multivariate Organization of Reference-Gene Expression Profiles Across 2D and 3D Culture Conditions

#### 3.9.1. Principal Component Analysis of Candidate Reference-Gene Profiles

**Figure 6:**
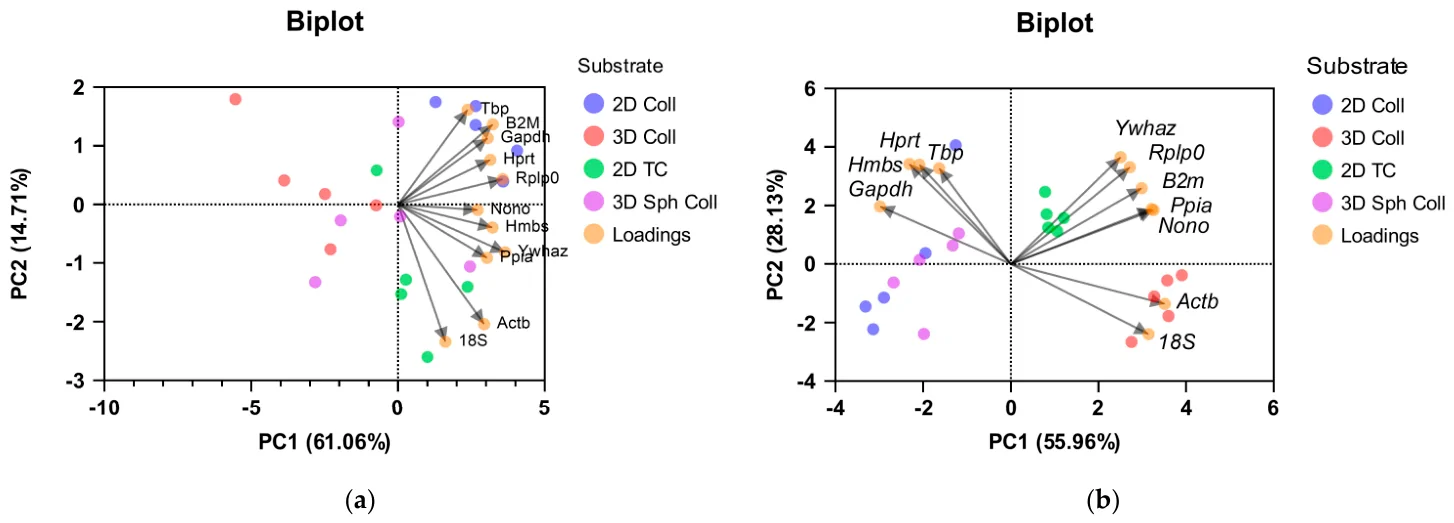
Principal component analysis of candidate reference-gene Cq profiles in (**a**) AML12 and (**b**) 3T3-L1 cells. Biplots display the distribution of samples according to culture condition, along with the magnitude and direction of the loadings of the 11 candidate reference genes. PC1 and PC2 explained 61.06% and 14.71% of the total variance in AML12 cells and 55.96% and 28.13% in 3T3-L1 cells, respectively. PCA was performed on standardized Cq values; n = 20 samples per cell line.

PCA revealed a condition-dependent organization of the overall Cq profile of the candidate reference genes in both cell lines (Figure 6). In AML12 cells, PC1 and PC2 accounted for 61.06% and 14.71% of the total variance, respectively, explaining 75.77% cumulatively. Sample separation occurred predominantly along PC1, with 2D Coll and 3D Coll occupying opposite regions, whereas Sph Coll and 2D TC showed more intermediate distributions. All 11 candidate genes loaded in the same direction on PC1, indicating a largely coordinated pattern of variation across the reference-gene panel. PC2 captured additional gene-specific structure: *B2m*, *Gapdh*, *Hprt*, *Rplp0*, and *Tbp* showed positive loadings, whereas *18S*, *Actb*, *Hmbs*, *Ppia*, and *Ywhaz* loaded in the opposite direction. *Nono* was positioned close to zero.

In 3T3-L1 cells, PC1 and PC2 explained 55.96% and 28.13% of the total variance, respectively, accounting for 84.09% cumulatively (Figure 6b). Sample scores showed a pronounced condition-associated structure: 2D Coll and Sph Coll samples were positioned predominantly toward negative PC1 values, whereas 3D Coll and 2D TC shifted toward positive PC1 values and were further separated along PC2. In contrast to AML12, the candidate reference genes separated into two opposing groups already along PC1. *18S*, *Actb*, *B2m*, *Nono*, *Ppia*, *Rplp0*, and *Ywhaz* showed positive loadings, whereas *Gapdh*, *Hmbs*, *Hprt*, and *Tbp* loaded in the opposite direction. Accordingly, the positive PC1 position of 3D Coll was associated with higher Cq values for the first group and lower Cq values for the second.

#### 3.9.2. Hierarchical Clustering of Candidate Reference-Gene Profiles

**Figure 7:**
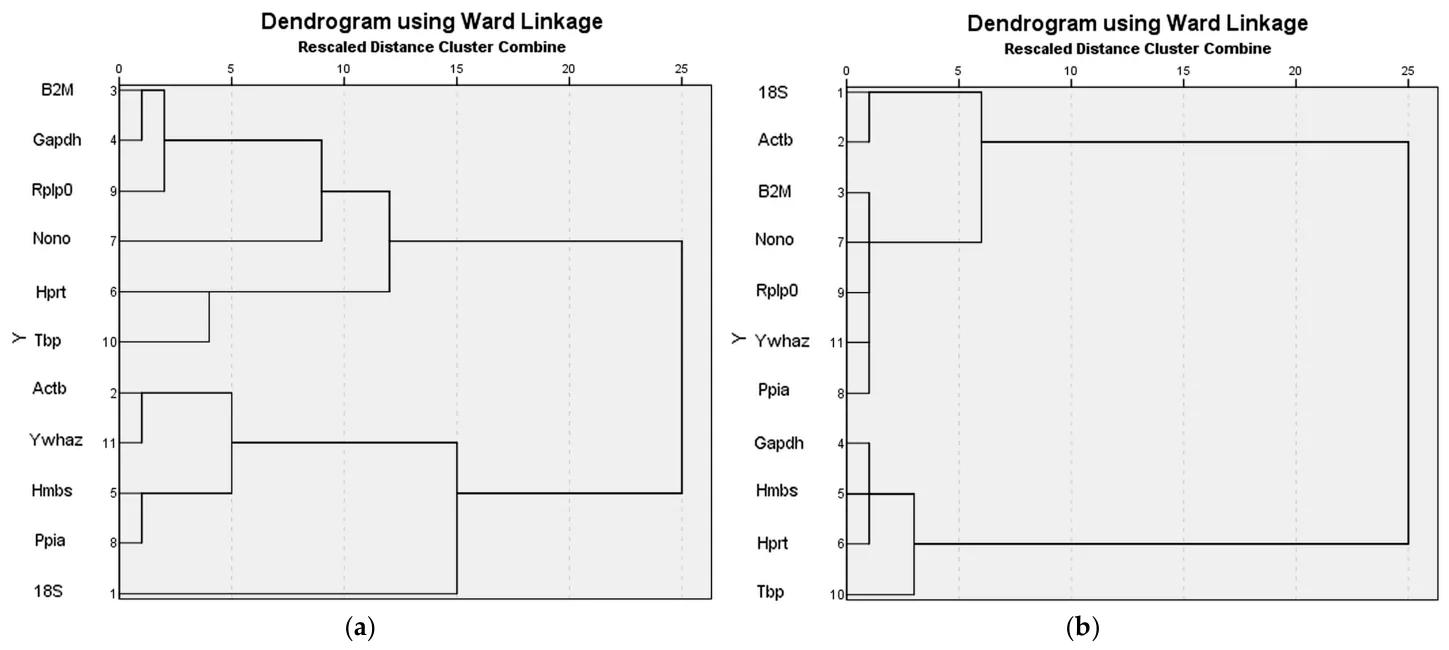
Hierarchical clustering of candidate reference-gene Cq profiles in AML12 and 3T3-L1 cells. Hierarchical clustering was performed on standardized Cq profiles using Ward’s linkage and squared Euclidean distance in (**a**) AML12 and (**b**) 3T3-L1 cells. The dendrograms illustrate similarities in condition-dependent Cq variation among the 11 candidate reference genes across 20 samples per cell line.

Hierarchical clustering of the standardized Cq profiles further demonstrated a cell line-specific organization of the candidate reference genes (Figure 7). In accordance with the PCA results in AML12 groups, two major clusters were identified: *B2m*, *Gapdh*, *Rplp0*, *Nono*, *Hprt*, and *Tbp* formed one cluster, whereas *18S*, *Actb*, *Ywhaz*, *Hmbs*, and *Ppia* grouped separately.

In 3T3-L1 cells, the clustering pattern differed from AML12 cells, but reproduced the same major structure as established in PCA, grouping *Gapdh*, *Hmbs*, *Hprt*, and *Tbp* separately from *18S*, *Actb*, *B2m*, *Nono*, *Rplp0*, *Ywhaz*, and *Ppia*, with particularly close clustering of *18S* and *Actb*.

### 3.10. Normalization-Dependent Changes in Target-Gene Expression and Statistical Interpretation

**Figure 8:**
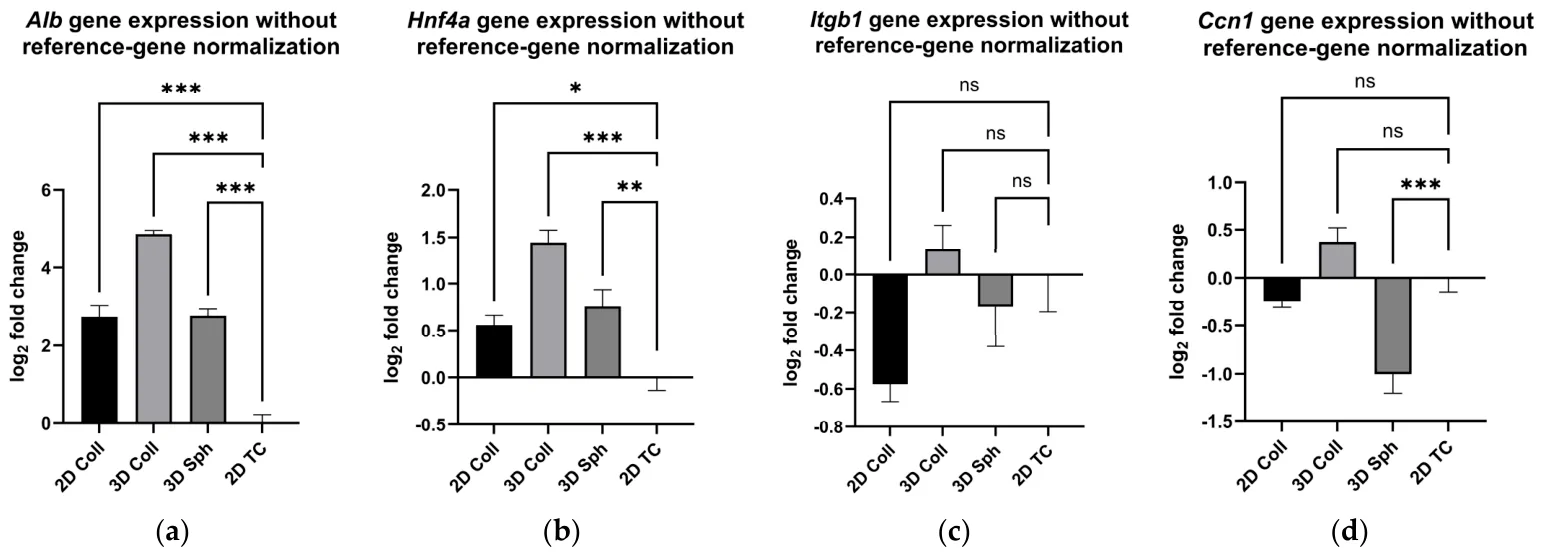

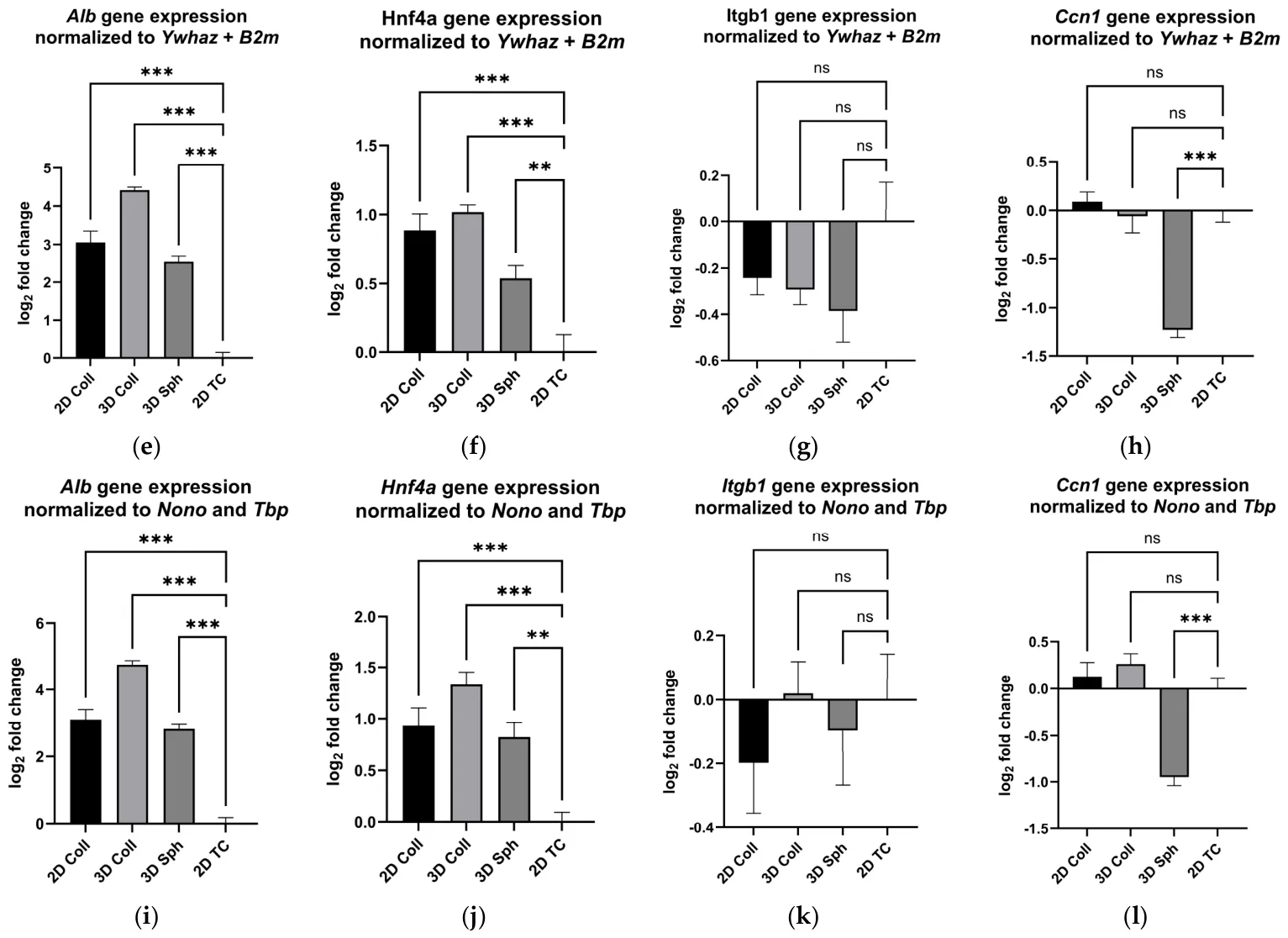
Normalization-dependent changes in target-gene expression across AML12 culture conditions. Relative expression of *Alb*, *Hnf4a*, *Itgb1*, and *Ccn1* was compared across 2D collagen (2D Coll), 3D collagen (3D Coll), collagen spheroid (Sph Coll), and 2D tissue-culture control (2D TC) conditions without reference-gene normalization and after normalization using two selected reference-gene pairs: (**a**–**d**) target-only relative expression without reference-gene normalization; (**e**–**h**) normalization to the NormFinder/geNorm-supported *Ywhaz* + *B2m* pair; and (**i**–**l**) normalization according to the BestKeeper—CV evaluated most stable pair, *Nono* + *Tbp*. Target-only relative expression was efficiency-corrected and calibrated to the 2D TC group without division by a reference-gene normalization factor. For reference-gene normalization, efficiency-corrected target quantities were divided by the geometric mean of the indicated reference-gene pair. Values are presented as log_2_ (fold change) relative to 2D TC; the horizontal line at 0 indicates no change relative to the calibrator. Data are presented as mean ± SD (n = 5 biological replicates). Statistical comparisons were performed separately for each target gene using ordinary one-way ANOVA followed by Dunnett’s multiple-comparisons test versus 2D TC on log_2_-transformed values. * *p* < 0.05, ** *p* < 0.01, *** *p* < 0.001; ns, not significant.

As a final validation step, the selected reference-gene panels were applied to the biologically informative target genes to determine how the choice of normalization factor affected the magnitude, pattern, and statistical interpretation of condition-dependent expression. The resulting normalized profiles were compared with the corresponding efficiency-corrected target-only profiles, used here as a common reference for assessing normalization-induced changes rather than as a definitive measure of true expression. In AML12 cells, the target-only profile showed increased *Alb* and *Hnf4a* expression across the collagen-based conditions, no significant changes in *Itgb1*, and a selective decrease in *Ccn1* in Sph Coll (Figure 8a–d). Normalization with either *Ywhaz* + *B2m* or *Nono* + *Tbp* largely preserved these condition-dependent expression patterns, although some differences in effect magnitude and significance level were observed, particularly for *Hnf4a* (Figure 8e–l). Thus, both selected two-gene panels reproduced the main biological pattern evident before reference-gene normalization.

**Figure 9:**
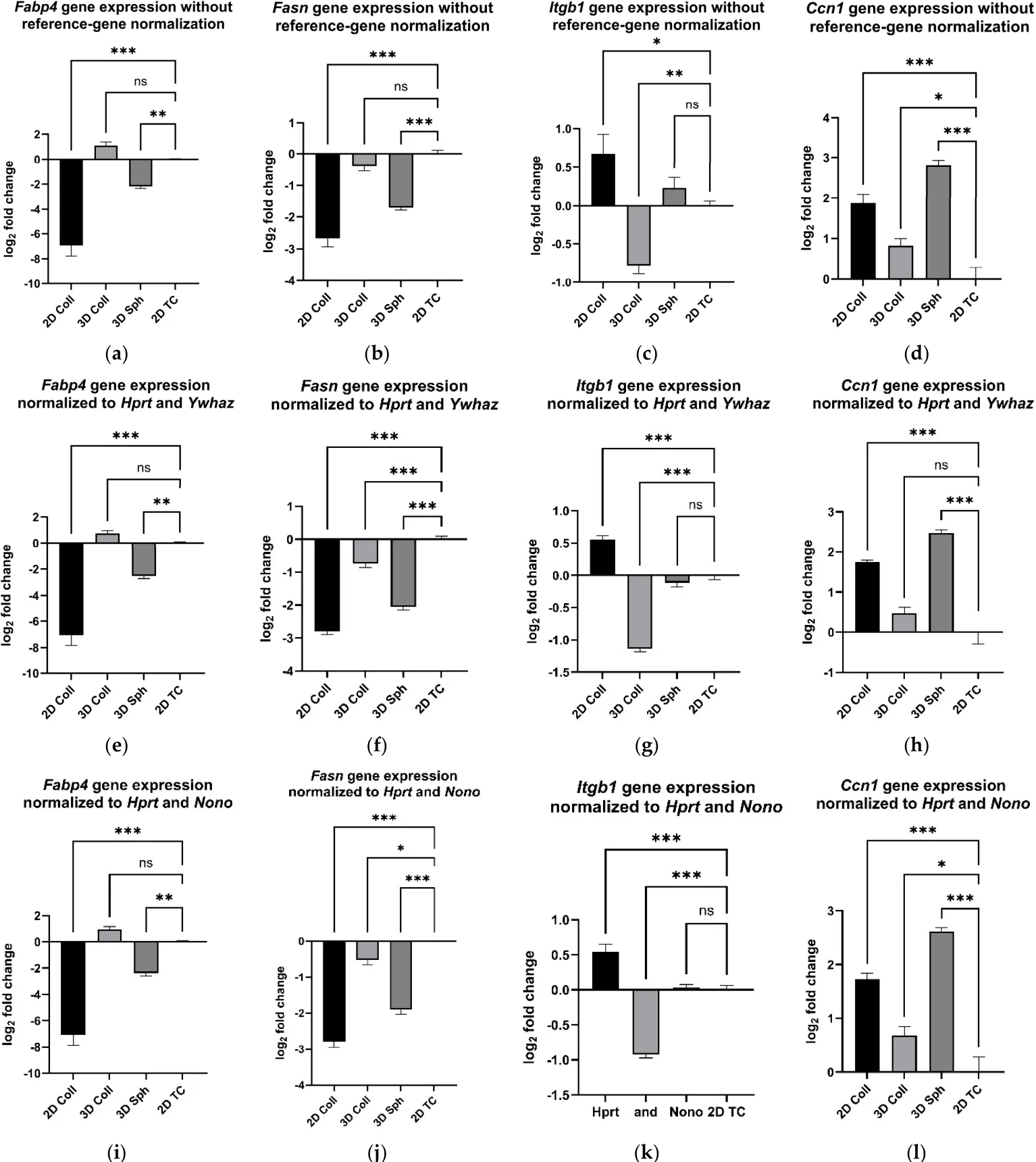

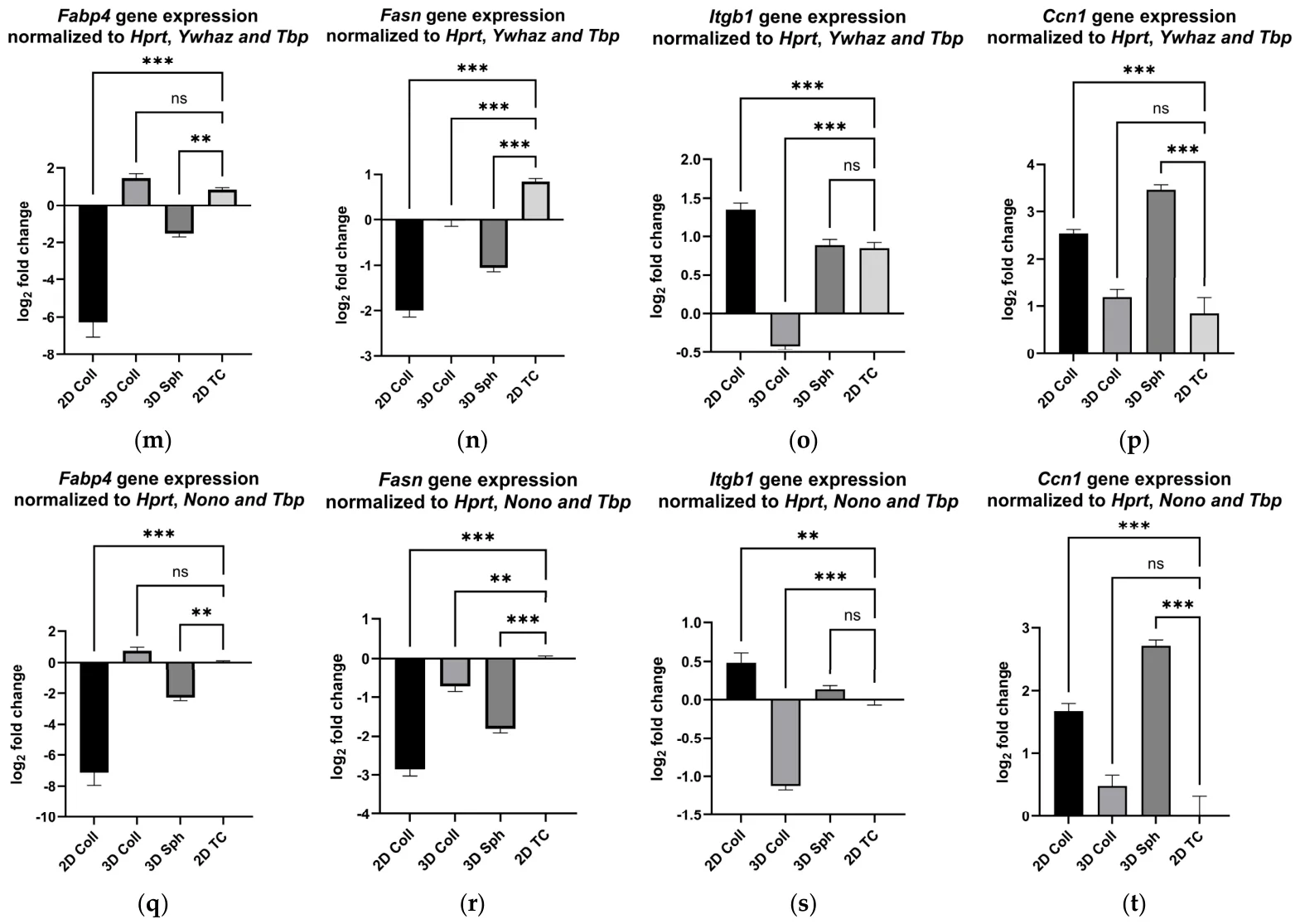
Normalization-dependent changes in target-gene expression across 3T3-L1 culture conditions. Relative expression of *Fabp4*, *Fasn*, *Itgb1*, and *Ccn1* was compared across 2D collagen (2D Coll), 3D collagen (3D Coll), collagen spheroid (Sph Coll), and 2D tissue-culture control (2D TC) conditions without reference-gene normalization and after normalization using selected reference-gene panels: (**a**–**d**) target-only relative expression without reference-gene normalization; (**e**–**h**) normalization to the BestKeeper SD-derived *Hprt* + *Ywhaz* pair; (**i**–**l**) normalization to the BestKeeper CV-derived *Hprt* + *Nono* pair; (**m**–**p**) normalization to the three-gene *Hprt* + *Ywhaz* + *Tbp* panel and (**q**–**t**) *Hprt* + *Nono* + *Tbp*. Target-only relative expression was efficiency-corrected and calibrated to the 2D TC group without division by a reference-gene normalization factor. For reference-gene normalization, efficiency-corrected target quantities were divided by the geometric mean of the indicated reference genes. Values are presented as log_2_ (fold change) relative to 2D TC; the horizontal line at 0 indicates no change relative to the calibrator. Data are presented as mean ± SD (n = 5 biological replicates). Statistical comparisons were performed separately for each target gene using ordinary one-way ANOVA followed by Dunnett’s multiple-comparisons test versus 2D TC on log_2_-transformed values. * *p* < 0.05, ** *p* < 0.01, *** *p* < 0.001; ns, not significant.

In 3T3-L1 cells, the target-only data showed pronounced condition-dependent changes in all four target genes (Figure 9a–d). Normalization with the *Hprt* + *Ywhaz* and *Hprt* + *Nono* pairs largely preserved the direction and overall pattern of these responses, although differences in effect magnitude and statistical significance remained, particularly for *Fasn* and *Itgb1* (Figure 9e–l). Addition of *Tbp* to the *Hprt* + *Ywhaz* pair did not further improve agreement with the target-only profile and introduced additional changes in the magnitude and/or statistical interpretation of some responses (Figure 9m–p). Thus, the greater target-gene independence of the three-gene BestKeeper Index did not translate into improved normalization performance.

## 4. Discussion

Previous work from our group on RT-qPCR normalization in 3T3-L1 adipocyte models has emphasized reference-gene validation under experimental conditions associated with substantial changes in cellular and metabolic status [14,15,36]. In 3T3-L1 adipocytes, we demonstrated that differentiation stage and treatment with lipid-modulating factors can markedly affect the expression of traditionally used internal controls [36]; whereas, in a subsequent model, extracellular matrix composition, differentiation stage, and the interaction between these two factors exerted gene-specific effects on the stability of candidate reference genes [15]. *Actb*, *18S*, and *Gapdh* were particularly sensitive to the experimental conditions, although the specific pattern of instability depended on the type of experimental perturbation.

The present study was designed differently. Within each cell line, the experimental groups were matched as closely as possible with respect to cell density, culture duration, and culture-medium composition. The corresponding 2D and 3D models were based on the same batch of collagen, with the same collagen concentration used in the 3D models, and 3T3-L1 cells were analyzed at the same stage of adipogenic differentiation. In this way, the influence of differences in metabolic maturity and additional experimental interventions was minimized, leaving culture conditions as the main source of biological variation. All stages of the RT-qPCR analysis were performed under standardized conditions and in accordance with MIQE principles, with the aim of maximizing control over technical sources of variability [3,37].

Under this controlled design, the candidate reference genes expectedly showed high overall expression stability. In AML12 cells, all 11 candidates (*18S*, *Actb*, *B2m*, *Gapdh*, *Hmbs*, *Hprt*, *Nono*, *Ppia*, *Rplp0*, *Tbp*, and *Ywhaz*) met the predefined screening criteria, whereas in 3T3-L1 cells the same was true for eight of the eleven candidates (*B2m*, *Hmbs*, *Hprt*, *Nono*, *Ppia*, *Rplp0*, *Tbp*, and *Ywhaz*).

The evaluation was conducted using different methodological approaches and more restrictive screening criteria, selected in accordance with the relatively homogeneous nature of the cell models. For geNorm, M < 1.0 was used instead of the classical threshold of M < 1.5, while M < 0.5 was considered indicative of particularly high stability. This interpretation was based on the qBase framework, according to which stable reference genes in relatively homogeneous sample panels typically show M < 0.5; whereas, in more heterogeneous panels, such as the 2D and 3D culture approaches applied here, acceptable values may reach approximately 1.0 [34]. For NormFinder, a stability value < 0.5 was applied as a literature-based screening benchmark [35], whereas for BestKeeper, SD < 1 Cq was used, supplemented by a more stringent assessment of CV. These thresholds were not regarded as universal boundaries between “stable” and “unstable” genes, but rather as a means of achieving higher discriminatory resolution during the initial screening.

Thus, the initial analysis identified a relatively broad set of potentially suitable genes for the subsequent selection of a normalization panel. At the same time, it again demonstrated the limited reliability of traditionally used single internal controls in 3T3-L1 models: *Actb*, *18S*, and *Gapdh* did not meet all of the applied criteria, in agreement with our previous observations [14,15,36]. For *Actb* and *18S*, the present finding is particularly informative because it was obtained using new primer pairs and an independently purchased 3T3-L1 cell line. Reproducibility across a different primer set and cell batch reduces the likelihood that the observed instability is assay- or batch-specific and further supports the context-dependent sensitivity of these genes in 3T3-L1 cells.

In addition to low overall variability, a suitable reference gene should remain as independent as possible of the experimental factor and, accordingly, should not systematically follow the condition-dependent changes in biologically regulated target genes. Otherwise, normalization may reduce or amplify the true target response [38,39]. A similar discrepancy between favorable algorithmic stability and a directly detected condition effect has also been reported in other cell models; for example, in MCF-7 cells, GAPDH was highly ranked by NormFinder but showed significant passage-dependent changes upon direct statistical testing [40].

BestKeeper provides an additional perspective from which this issue can be examined. In the original algorithm, the BestKeeper Index can be correlated both with individual candidate reference genes and with target genes, thereby allowing assessment of whether they follow similar expression profiles [23]—an analysis that, in contemporary applications of the method, is often given less emphasis than SD/CV and HKG-BestKeeper Index correlations. A high correlation of a given HKG with the BestKeeper Index primarily reflects its concordance with the overall reference-gene profile, but does not in itself demonstrate that this profile is biologically independent of the experimental response. Our results clearly illustrate this distinction.

For the subsequent evaluation, we used target genes reflecting both cellular functional identity (*Alb*, *Hnf4a*; *Fabp4*, *Fasn*) and the response to culture architecture (*Itgb1*, *Ccn1*) to determine whether stable reference genes remained independent of biologically regulated variation. In AML12 cells, *Ywhaz* was both the highest-ranked individual candidate according to NormFinder and the gene showing the strongest correlation with the BestKeeper Index, yet direct Pearson analysis with the target genes revealed strong associations of *Ywhaz* with *Itgb1* (r = 0.78) and *Hnf4a* (r = 0.82). Similar target-specific correlations were observed for other algorithmically stable candidates, including *Ppia* with *Alb* and *Hnf4a* (r = 0.84 and 0.86, respectively).

The contrast was even more pronounced in 3T3-L1 cells. *B2m*, *Nono*, *Ppia*, *Rplp0*, and *Ywhaz* showed strong concordance with the recalculated BestKeeper Index (r = 0.889–0.962), yet the same candidates displayed multiple moderate-to-strong correlations with target genes. For example, *B2m* correlated positively with *Itgb1* and *Ccn1* (r = 0.76 and 0.74, respectively) and negatively with *Fabp4* and *Fasn* (r = −0.70 and −0.70, respectively), whereas *Nono* showed r = 0.92 with *Itgb1* and r = −0.70 with *Fabp4*. Thus, selecting candidates that most closely follow the overall BestKeeper profile increases the internal concordance of the normalization set, but does not guarantee its independence from target-gene variation.

The significance of such target-reference dependence is also supported by independent statistical studies from other authors. Cui et al. [41] showed that reference- and target-gene Cq values can be systematically related and that different targets may exhibit different regression coefficients relative to the same reference set. The authors demonstrated that such relationships can affect both the calculated fold change and the statistical conclusions following standard ΔCq normalization. Although their analysis focused on mathematical bias introduced during normalization, their findings provide additional support for evaluating reference-target covariance as a separate component in the selection of a normalization factor.

The discrepancy observed among the different assessments in our study raised the question of whether high inter-reference concordance reflects only stable expression or, at least in part, a coordinated response to the culture condition. The LMM supported the latter interpretation, identifying a significant culture-condition effect for 9 of the 11 candidates in AML12 cells and for all 11 candidates in 3T3-L1 cells. Only *18S* and *Nono* in AML12 cells showed no significant dependence on culture condition.

Therefore, 3D culture should not be regarded as a technical variation in the standard 2D model. Culture architecture itself, together with the matrix context, can systematically alter reference-gene expression. Consequently, neither candidates validated under conventional 2D conditions nor apparently stable genes identified by a single algorithm should automatically be considered suitable for 3D systems. Model- and substrate-specific validation based on more than one criterion is required [18,27,28].

According to MIQE and the updated MIQE 2.0 recommendations, both the selection and the number of reference genes should be experimentally justified for the specific model [3,37]. Therefore, the next step was to determine the required size of the normalization panel. In both cell lines, geNorm pairwise-variation analysis showed V2/3 < 0.15, indicating that the addition of a third reference gene would not substantially improve the normalization factor according to this criterion. However, the different stability approaches did not consistently identify the same optimal pair, consistent with previous observations that algorithms based on different mathematical principles may produce partly divergent reference-gene rankings [42]. This raised the practical question of which of the several algorithmically supported combinations would be most appropriate for actual normalization.

For this purpose, a separate BestKeeper Index was constructed for each preselected pair, and its association with the biologically informative target-gene panel was evaluated. This approach was used not as a substitute for conventional stability ranking, but as an additional check of whether the high internal concordance of a given pair was accompanied by limited co-variation with target-gene expression. The results again showed that high internal concordance was not always accompanied by limited target-gene co-variation. For example, the *Ywhaz* + *B2m* combination in AML12 cells, identified as the most stable by NormFinder and geNorm, showed high inter-reference concordance with the BK Index, but this index remained strongly associated with *Hnf4a* (r = 0.725) and *Itgb1* (r = 0.791). In 3T3-L1 cells, this contrast was even more pronounced: *Nono* + *Ywhaz* and *Ywhaz* + *Rplp0* reached r = 0.947–0.987 against their respective BestKeeper indices, but the indices correlated significantly with all four target genes (r = 0.490–0.865). Vandesompele et al. [21] already noted that coordinately changing genes can maintain a relatively constant expression ratio and therefore be assessed as highly stable. Chervoneva et al. [22] later showed that the correlation structure among candidates can influence the selection of the normalization set, while Cui et al. [41] demonstrated that dependence between reference- and target-gene expression can affect both fold-change estimates and the statistical interpretation after normalization.

In the present study, Pearson correlations generated using the BestKeeper tool, as well as the individual comparisons performed in GraphPad, were not interpreted as evidence of direct co-regulation, because they may also reflect a shared response to the different 2D and 3D conditions. Instead, they were interpreted together with the LMM, PCA, and hierarchical clustering results. PCA and hierarchical clustering allowed us, independently of the conventional ranking, to assess the extent to which genes within a given combination followed similar or distinct multivariate profiles and, accordingly, whether they were more likely to be redundant or complementary.

In AML12 cells, integration of the results from the analyses described above identified *Tbp* + *Nono*—the pair with the lowest CV according to BestKeeper—as the most balanced normalization panel. *Nono* was one of only two candidates without a significant effect of culture condition in the LMM analysis, whereas for *Tbp*, although this effect was statistically significant, its magnitude remained relatively moderate (F = 6.606; *p* < 0.01). PCA and hierarchical clustering further showed that the two genes did not follow identical multivariate expression profiles. They belonged to the same major cluster but did not form an immediately linked pair, while along PC2 Tbp showed a clearly positive loading and *Nono* was positioned close to the neutral axis, suggesting limited redundancy within the overall coordinated expression shift in AML12 cells. At the same time, the combined BestKeeper Index for *Tbp* + *Nono* was not significantly associated with *Alb*, *Hnf4a*, or *Ccn1*, with a significant relationship retained only with *Itgb1* (r = 0.694). This interpretation was also supported by the direct pairwise correlation analysis in GraphPad Prism, in which *Nono* showed the most consistently limited co-variation across the entire target panel, whereas *Tbp* showed particularly weak associations with the hepatocyte identity markers *Alb* and *Hnf4a*. Taken together, these characteristics indicate that *Tbp* + *Nono* combines acceptable stability and low variability with partially distinct multivariate profiles and relatively limited target-gene co-variation, making it a more balanced choice than, for example, the pair identified by NormFinder and geNorm (*Ywhaz* + *B2m*), which showed higher internal concordance but more pronounced co-variation with biologically regulated target-gene expression.

In 3T3-L1 cells, the pattern was even more pronounced. Culture condition had a significant effect on all 11 candidate reference genes, with the strongest effects observed for *Actb* and *18S*. This was consistent with the lower stability of the same candidates in the classical analyses and with a greater disruption of reference-gene homeostasis associated with changes in culture architecture. Hierarchical clustering and PCA revealed a more heterogeneous structure with clearly defined components, with the first two components explaining 84.09% of the variance. However, unlike in AML12 cells, the candidates were distributed in opposite directions along PC1. Thus, in 3T3-L1 cells, the experimental effect did not represent only a coordinated shift in the entire HKG profile, but also included distinct and partially opposing gene-specific patterns.

The classical geNorm and NormFinder analyses in this cell line identified *Nono* + *Ywhaz* and *Ywhaz* + *Rplp0* as the leading pairs. The multivariate analyses showed that these genes belonged to the same component and cluster and followed similar expression profiles, while the direct HKG-target correlation analysis revealed similar co-variation with the biologically regulated targets. Particular attention should be given to *Ywhaz*, which statistically performed as one of the most stable candidates, either alone or in combination, but encodes 14-3-3ζ, a protein with an established role in 3T3-L1 adipogenesis, including adipocyte differentiation, chromatin accessibility, and the regulation of metabolic processes and *Pparg* expression. This does not disqualify *Ywhaz* as a reference gene, because validation should be based primarily on its behavior in the specific experimental model, but it does call into question its automatic inclusion in the final normalization panel solely on the basis of a high stability rank. When the second gene in the pair follows a similar condition-dependent expression profile, the combined normalization factor gains greater internal concordance, but not necessarily greater biological independence. In the present data, this limitation is supported by the significant correlations between the BestKeeper indices of these pairs and some of the analyzed target genes, indicating that high concordance between reference genes may coincide with partial tracking of biologically regulated expression changes.

In contrast to the combinations evaluated by geNorm and NormFinder, the BestKeeper SD and CV rankings identified pairs comprising genes with distinct expression profiles. *Hprt* + *Ywhaz* and *Hprt* + *Nono* linked candidates from the two major clusters in the hierarchical analysis and occupied opposite positions along PC1: *Hprt* was located in the negative, whereas *Ywhaz* and *Nono* were positioned in the positive direction of the loading space. The direct HKG-target analysis likewise showed clearly distinct behavior of the individual components: *Hprt* correlated negatively with *Itgb1*/*Ccn1* and positively with *Fabp4*/*Fasn*, whereas *Ywhaz*, and particularly *Nono*, followed the opposite profile. Thus, these BestKeeper-derived combinations paired genes with more complementary, rather than parallel, condition-dependent variation.

Accordingly, the pair-specific BestKeeper indices showed more limited dependence on target-gene expression: for *Hprt* + *Ywhaz*, a significant association remained only with *Ccn1* (r = 0.488), whereas for *Hprt* + *Nono* there was no association with *Fabp4* or *Fasn*, with only moderate relationships with *Itgb1* and *Ccn1* (r = 0.483 and 0.465, respectively). Taken together, these characteristics better conform to the principle that the normalization factor should remain independent of biologically regulated expression and indicate that PCA, hierarchical clustering, and direct HKG-target correlation analysis can substantially support the selection of complementary reference-gene combinations beyond classical stability ranking.

The final functional validation of the selected normalization panels was performed by applying them to the biologically informative panel of target genes and comparing the resulting expression profiles with the efficiency-corrected target-only profiles, i.e., without normalization to a reference gene (Figure 8 and Figure 9). Target-only expression was not considered a “true” or reference result, but rather a conditional benchmark for assessing sensitivity to the choice of normalization factor and the extent to which it altered the direction, magnitude, or statistical interpretation of the experimental response. The main figures present the combinations selected on the basis of the integrated information from stability analyses, dependence on culture condition, associations with target genes, and multivariate expression profiles, whereas Supplementary Figures S3 and S4 extend the comparison to alternative normalization strategies, including traditionally used single reference genes, pairs of candidates with similar expression behavior, and *Ywhaz* as a single normalizer. Supplementary Figure S3, for example, includes different single- and two-gene normalization strategies for AML12 cells. Notably, the reference-gene combinations that successfully passed the multistep validation reproduced, after normalization, target-gene expression patterns that most closely resembled those observed in the conditional target-only profiles, whereas other normalizers, although conventionally stable, altered the magnitude of the response to a greater extent and, in some cases, its statistical and biological interpretation.

Because of the higher variability in reference-gene expression and the more pronounced condition-dependent reorganization in 3T3-L1 cells, an additional evaluation using three-gene normalization panels was also performed. This step was not intended to revise the geNorm conclusion that two reference genes were sufficient, but rather to assess whether the addition of a third, more independent candidate could limit the influence of shared variation among the components of the two-gene panels on biological interpretation. For this purpose, *Tbp* was added to selected two-gene combinations. Its selection was based on its specific profile: *Tbp* met the stability criteria of all applied classical algorithms, but, unlike most of the other stable candidates, showed weak and statistically non-significant correlations with all four target genes (r = −0.25 to 0.22).

When *Tbp* was added to the two normalization pairs previously characterized as relevant to this experimental context, the corresponding three-gene BestKeeper indices showed no significant association with any of the target genes. However, this greater target-gene independence did not translate into a more favorable expression profile after normalization, as the three-gene panels did not reproduce the target-only pattern better than the corresponding two-gene combinations. Thus, the functional validation confirmed the conclusion of the geNorm pairwise-variation analysis that inclusion of a third reference gene provided no additional advantage in the present experimental model.

The results of the present study support a multistep approach to the selection of a normalization panel, in which individual methods provide distinct but complementary information. Initial expression stability should be evaluated using algorithms that examine different characteristics of the data [42]: BestKeeper correlation analysis characterizes the concordance of candidates with the overall reference-gene profile, geNorm pairwise variation determines the required number of reference genes, while direct evaluation of HKG-target relationships, complemented by analysis of condition dependence and multivariate expression patterns, allows stability, redundancy, and independence from biologically regulated expression to be distinguished. Final validation through actual normalization of target genes demonstrates that these characteristics are not interchangeable and that even algorithmically stable panels can lead to different biological interpretations.

**Figure 10:**
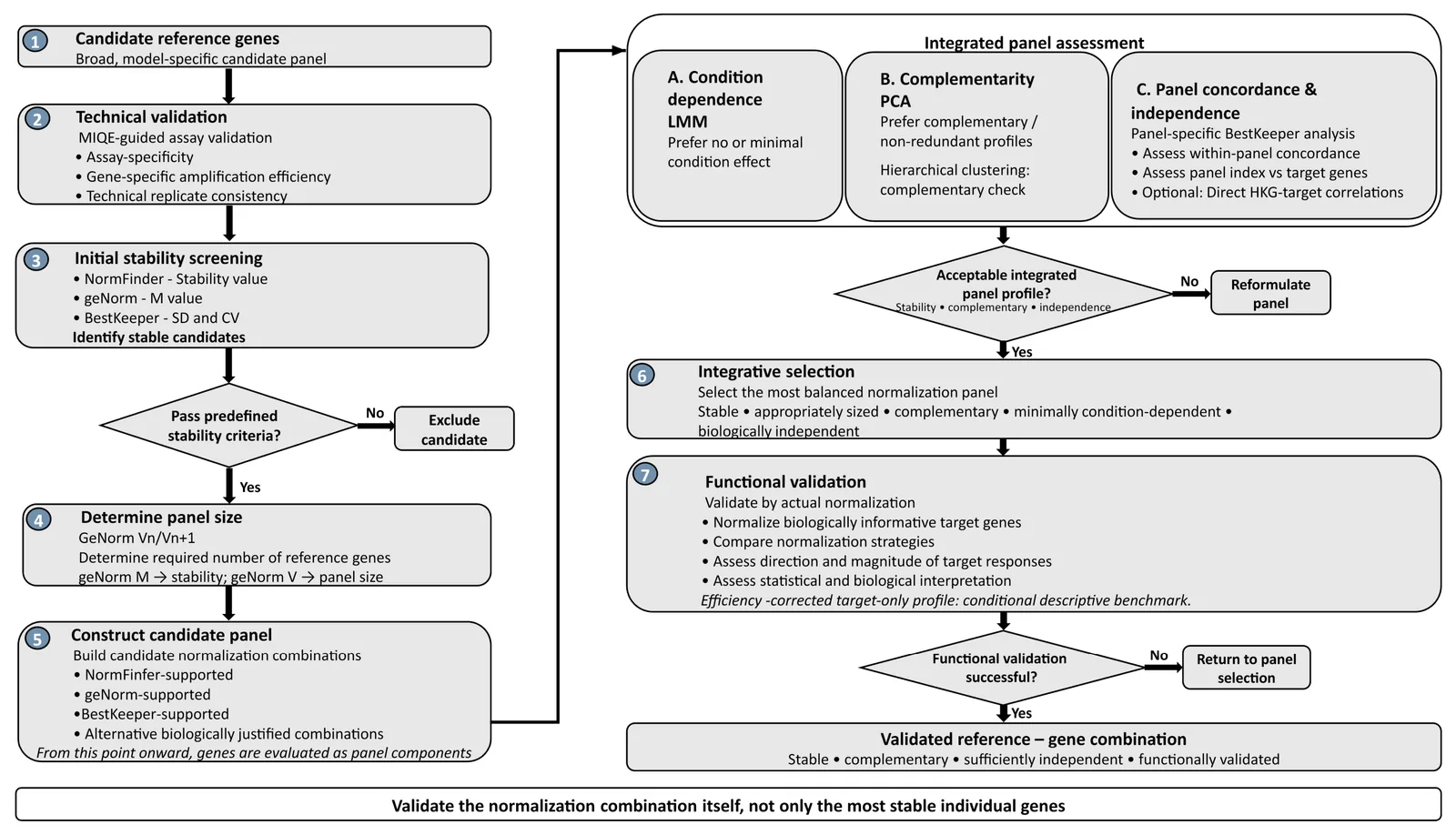
Multistep strategy for selecting reliable reference-gene combinations for RT-qPCR normalization. The workflow integrates initial stability screening, determination of normalization-panel gene size, assessment of condition dependence, expression complementarity, and biological independence, followed by functional validation through normalization of biologically informative target genes. Reference-gene panels that do not meet the integrated criteria or fail functional validation are reformulated and reassessed. The final goal is to identify a reference-gene combination that is not only stable but also complementary, sufficiently independent, and functionally validated.

Based on these findings, the proposed practical workflow for selecting and validating reference-gene combinations is summarized in Figure 10.

The present study should be interpreted in the context of its specific experimental design. The analysis was conducted in two cell lines and under defined 2D and 3D culture conditions; therefore, future application of the proposed strategy in other models will be important for assessing its broader generalizability. The five biological replicates used per condition enabled consistent comparison among the different approaches, although larger sample sizes could further improve the precision of weaker association estimates. Functional validation in the present study was deliberately based on a panel of biologically informative target genes and clearly demonstrated the sensitivity of biological interpretation to the choice of normalization panel. In future studies, inclusion of a broader range of target genes and independent functional readouts would allow the behavior of normalized gene expression to be linked even more directly to cellular phenotype and function. This would extend, rather than replace, the present multistep validation framework and would allow its applicability to more complex 2D/3D experimental systems to be further evaluated.

## 5. Conclusions

The results of the present study showed that, when comparing 2D and 3D models, the main challenge is not the identification of stable reference genes, but determining which combination of already stable candidates provides the most reliable normalization. All 11 HKGs in AML12 cells passed the initial screening, whereas in 3T3-L1 cells only *Actb*, *18S*, and *Gapdh* were excluded. After geNorm indicated that two reference genes were sufficient, the differences among the algorithmically preferred pairs raised the question of which combination was actually most appropriate for normalization. The present study extends classical stability assessment by distinguishing three different characteristics—expression stability, inter-reference concordance, and biological independence—and by using PCA and hierarchical clustering to evaluate expression redundancy among already stable candidates. Integration of these data with condition dependence, HKG-target relationships, and final validation through the normalization itself showed that a reliable panel should combine not only stable and functionally distinct genes, but also genes that are expression-nonredundant and sufficiently independent. Thus, the proposed approach shifts the focus from selecting the “most stable” genes toward experimental validation of the reference-gene combination itself and of its effect on biological interpretation.

## Figures and Tables

**Table 1:** Sequences of selected housekeeping and target genes used in RT-qPCR.

Abbreviation	Full Name	Forward Primer	Reverse Primer	Amplicon Size (bp)
*18S* NR_003278.3	18S ribosomal RNA	CGTTCTTAGTTGGTGGAGCG	AACGCCACTTGTCCCTCTAA	127
*Actb* NM_007393.5	β-actin	CTCCTAGCACCATGAAGATCAA	GCAGCTCAGTAACAGTCCGC	187
*Gapdh* NM_001289726.2	Glyceraldehyde-3-phosphate dehydrogenase	CCCACTCTTCCACCTTCGAT	CTTGCTCAGTGTCCTTGCTG	181
*B2m* NM_009735.3	Beta-2 microglobulin	TGTATGCTATCCAGAAAACCCCT	TTTCAATGTGAGGCGGGTGG	117
*Ppia* NM_008907.2	Peptidylprolyl isomerase A	CTGGACCAAACACAAACGGT	TGCCCGCAAGTCAAAAGAAA	208
*Hmbs* NM_001110251.1	Hydroxymethylbilane synthase	CCTGAAGGATGTGCCTACCA	CCACTCGAATCACCCTCATCT	175
*Rplp0* NM_007475.5	Ribosomal protein, large, P0	GAAACTGCTGCCTCACATCC	AGGTCTTCTCGGGTCCTAGA	179
*Hprt* NM_013556.2	Hypoxanthine guanine phosphoribosyl transferase	ACAGGCCAGACTTTGTTGGA	ACTTGCGCTCATCTTAGGCT	150
*Nono* NM_001426221.1	Mus musculus non-POU domain-containing, octamer-binding protein	AAGGAGAGAGAACAGCCACC	TAACCTGGTGCTCATGACGT	184
*Tbp* NM_013684.3	TATA-box binding protein	AATTGTACCGCAGCTTCAAAAT	CAGTTGTCCGTGGCTCTCTT	152
*Ywhaz* NM_001356569.1	Tyrosine 3-monooxygenase/tryptophan 5-monooxygenase activation protein, zeta polypeptide	AGACGGAAGGTGCTGAGAAA	TTGTCATCACCAGCAGCAAC	211
*Itgb1* NM_010578.2	integrin beta 1 (fibronectin receptor beta)	GCTTAATTTGTGGAGGCAATGG	GACGCTAGACATGGACCAGT	112
*Ccn1* NM_010217.2	Cellular communication network factor 1	TGCTGTGAAGAGTGGGTTTGT	GGTGCCAAAGACAGGAAGCC	165
*Fabp4* NM_024406.4	Fatty acid-binding protein 4	AACTGGGCGTGGAATTCGAT	CCACCAGCTTGTCACCATCT	150
*Fasn* NM_007988.3	Fatty acid synthase	CTGAAGCCGAACACCTCTGT	GGGAATGTTACACCTTGCTCCT	218

**Table 2:** Correlation of candidate reference and target genes with the BestKeeper Index in AML12 cells.

BestKeeper Index vs.	*Hmbs*	*B2m*	*Hprt*	*Nono*	*Ppia*	*Rplp0*	*Tbp*	*Ywhaz*	*18S*	*Actb*	*Alb*	*Hnf4a*	*Itgb1*	*Ccn1*
*n*	20	20	20	20	20	20	20	20	20	20	20	20	20	20
coeff. of correlation (r)	0.734	0.841	0.778	0.692	0.811	0.883	0.550	0.967	0.555	0.854	0.588	0.792	0.789	0.466
*p*-value	0.001	0.001	0.001	0.001	0.001	0.001	0.012	0.001	0.011	0.001	0.006	0.001	0.001	0.039

*Gapdh* was not included in BestKeeper Index construction because BestKeeper v1 accepts a maximum of ten candidate reference genes. Although *Gapdh* fulfilled the predefined AML12 stability criteria, it showed the least favorable overall profile across the preceding stability analyses and was therefore omitted at this stage.

**Table 3:** Correlation of candidate reference and target genes with the BestKeeper Index in 3T3-L1 cells.

BestKeeper Index vs.	*Hmbs*	*B2m*	*Hprt*	*Nono*	*Ppia*	*Rplp0*	*Tbp*	*Ywhaz*	*Fabp4*	*Fasn*	*Itgb1*	*Ccn1*
*n*	20	20	20	20	20	20	20	20	20	20	20	20
coeff. of correlation (r)	0.243	0.846	0.271	0.746	0.747	0.918	0.312	0.942	−0.324	−0.351	0.601	0.687
*p*-value	ns	0.001	ns	0.001	0.001	0.001	ns	0.001	ns	ns	0.005	0.001

*Actb*, *18S*, and Gapdh were excluded before BestKeeper Index construction because they failed at least one criterion during the preceding stability screening. ns, not significant.

**Table 4:** Recalculated BestKeeper correlation analysis in 3T3-L1 cells after refinement of the reference-gene set.

BestKeeper Index vs.	*B2m*	*Nono*	*Ppia*	*Rplp0*	*Ywhaz*	*Fabp4*	*Fasn*	*Itgb1*	*Ccn1*
*n*	20	20	20	20	20	20	20	20	20
coeff. of correlation (r)	0.945	0.889	0.904	0.962	0.930	−0.619	−0.635	0.810	0.818
*p*-value	0.001	0.001	0.001	0.001	0.001	0.004	0.003	0.001	0.001

**Table 5:** Linear mixed-effects model analysis of culture-condition effects on candidate reference-gene Cq values in AML12 cells.

Gene	2D Coll	3D Coll	Sph Coll	TC Coll	F(3,12)	*p*
*18S*	13.018 ± 0.172 ^a^	12.890 ± 0.172 ^a^	13.142 ± 0.172 ^a^	13.120 ± 0.172 ^a^	0.427	0.738
*Actb*	17.896 ± 0.129 ^ab^	17.384 ± 0.129 ^b^	17.768 ± 0.129 ^ab^	18.150 ± 0.129 ^a^	5.087	0.017
*B2m*	22.780 ± 0.091 ^a^	22.022 ± 0.091 ^b^	22.060 ± 0.091 ^b^	22.294 ± 0.091 ^b^	16.281	<0.001
*Gapdh*	18.492 ± 0.102 ^a^	17.390 ± 0.102 ^b^	17.290 ± 0.102 ^b^	17.500 ± 0.102 ^b^	34.346	<0.001
*Hmbs*	26.456 ± 0.175 ^a^	25.330 ± 0.175 ^b^	26.114 ± 0.175 ^a^	26.282 ± 0.175 ^a^	8.796	0.002
*Hprt*	22.346 ± 0.064 ^a^	21.514 ± 0.064 ^b^	22.150 ± 0.064 ^a^	21.798 ± 0.064 ^c^	34.032	<0.001
*Nono*	23.796 ± 0.155 ^a^	23.366 ± 0.155 ^a^	23.380 ± 0.155 ^a^	23.558 ± 0.155 ^a^	1.822	0.197
*Ppia*	20.138 ± 0.097 ^bc^	19.500 ± 0.097 ^a^	19.950 ± 0.097 ^b^	20.368 ± 0.097 ^c^	18.958	<0.001
*Rplp0*	19.824 ± 0.098 ^a^	19.206 ± 0.098 ^b^	19.352 ± 0.098 ^b^	19.526 ± 0.098 ^ab^	8.578	0.003
*Tbp*	27.982 ± 0.116 ^a^	27.408 ± 0.116 ^b^	27.750 ± 0.116 ^ab^	27.448 ± 0.116 ^b^	6.606	0.007
*Ywhaz*	21.786 ± 0.109 ^a^	21.032 ± 0.109 ^b^	21.410 ± 0.109 ^ab^	21.616 ± 0.109 ^a^	10.715	0.001

Values are presented as estimated marginal means ± SE from linear mixed-effects models, with biological replicate specified as the subject for repeated measurements. Different superscript letters within a row indicate statistically significant differences among culture conditions after Bonferroni correction (*p* < 0.05); values sharing at least one letter do not differ significantly. Lower Cq values correspond to higher expression levels. For each condition, n = 5 biological replicates.

**Table 6:** Linear mixed-effects model analysis of culture-condition effects on candidate reference-gene Cq values in 3T3-L1 cells.

Gene	2D Coll	3D Coll	Sph Coll	TC Coll	F(3,12)	*p*
*18S*	11.740 ± 0.253 ^a^	15.894 ± 0.253 ^b^	12.034 ± 0.253 ^a^	11.960 ± 0.253 ^a^	59.015	<0.001
*Actb*	18.112 ± 0.111 ^ab^	20.894 ± 0.111 ^c^	17.804 ± 0.111 ^b^	18.596 ± 0.111 ^a^	147.572	<0.001
*B2m*	20.414 ± 0.170 ^a^	21.470 ± 0.170 ^bc^	20.824 ± 0.170 ^ac^	21.696 ± 0.170 ^b^	12.695	<0.001
*Gapdh*	19.374 ± 0.140 ^a^	17.408 ± 0.140 ^c^	18.716 ± 0.140 ^ab^	18.212 ± 0.140 ^b^	29.893	<0.001
*Hmbs*	27.708 ± 0.181 ^a^	26.514 ± 0.181 ^b^	27.556 ± 0.181 ^a^	27.252 ± 0.181 ^ab^	9.400	0.002
*Hprt*	25.948 ± 0.108 ^a^	25.088 ± 0.108 ^b^	25.498 ± 0.108 ^bc^	25.598 ± 0.108 ^ac^	12.795	<0.001
*Nono*	23.870 ± 0.130 ^a^	24.726 ± 0.130 ^b^	24.204 ± 0.130 ^ac^	24.500 ± 0.130 ^bc^	11.155	0.001
*Ppia*	20.772 ± 0.122 ^a^	21.498 ± 0.122 ^b^	20.374 ± 0.122 ^a^	21.354 ± 0.122 ^b^	18.077	<0.001
*Rplp0*	20.242 ± 0.128 ^a^	20.788 ± 0.128 ^bc^	20.264 ± 0.128 ^ac^	21.166 ± 0.128 ^b^	14.122	<0.001
*Tbp*	28.054 ± 0.135 ^ab^	27.590 ± 0.135 ^a^	28.466 ± 0.135 ^b^	28.354 ± 0.135 ^b^	9.512	0.002
*Ywhaz*	22.490 ± 0.136 ^a^	22.890 ± 0.136 ^ab^	22.508 ± 0.136 ^a^	23.088 ± 0.136 ^b^	6.398	0.008

Data are presented as estimated marginal means ± SE from linear mixed-effects models, with biological replicate specified as the subject for repeated measurements. Different superscript letters within a row indicate statistically significant differences among culture conditions in Bonferroni-adjusted pairwise comparisons (*p* < 0.05); groups sharing at least one letter do not differ significantly. Lower Cq values correspond to higher expression levels. For each condition, n = 5 biological replicates.

**Table 7:** Most stable two-gene combinations identified by different reference-gene stability approaches in AML12 and 3T3-L1 cells.

Stability Approach	AML12—Top-Ranked Two-Gene Set	3T3-L1—Top-Ranked Two-Gene Set
NormFinder	*B2m + Ywhaz* ^1^	*Nono + Ywhaz* ^1^
geNorm	*Ywhaz + B2m*	*Ywhaz + Rplp0*
BestKeeper—SD	*Rplp0 + Nono/18S* ^2^	*Hprt + Ywhaz*
BestKeeper—CV	*Tbp + Nono*	*Hprt + Nono*
Comparative ΔCq (Silver et al. [32])	*Rplp0 + Ywhaz*	*Ywhaz + Rplp0*
RefFinder	*Rplp0 + Ywhaz*	*Ywhaz + Rplp0*

Table note: ^1^ NormFinder reports a model-derived optimal two-gene combination rather than simply the two highest-ranked individual genes. ^2^ *Nono* and *18S* showed identical BestKeeper SD values after rounding (0.28 Cq).

**Table 8:** BestKeeper-based evaluation of the independence of pre-established reference-gene pairs from target-gene expression in AML12 cells.

Top Ranked Reference Gane/Pair	Gene 1 vs. BK Index, r	Gene 2 vs. BK Index, r	Target 1 *Alb*, r (*p*)	Target 2 *Hnf4a*, r (*p*)	Target 3 *Itgb*, r (*p*)	Target 4 *Ccn1*, r (*p*)
*Ywhaz* + *B2m*	r = 0.815 *p* = 0.001	r = 0.937 *p* = 0.001	r = 0.434 ns	r = 0.725 *p* = 0.001	r = 0.791 *p* = 0.001	r = 0.421 ns
*Rplp0* + *Nono*	r = 0.883 *p* = 0.001	r = 0.889 *p* = 0.001	r = 0.362 ns	r = 0.559 *p* = 0.010	r = 0.721 *p* = 0.001	r = 0.204 ns
*Rplp0* + *18S*	r = 0.670 *p* = 0.001	r = 0.908 *p* = 0.001	r = 0.387 ns	r = 0.686 *p* = 0.001	r = 0.822 *p* = 0.001	r = 0.424 ns
*Tbp* + *Nono*	r = 0.757 *p* = 0.001	r = 0.860 *p* = 0.001	r = 0.163 ns	r = 0.358 ns	r = 0.694 *p* = 0.001	r = 0.385 ns
*Rplp0* + *Ywhaz*	r = 0.946 *p* = 0.001	r = 0.953 *p* = 0.001	r = 0.537 *p* = 0.015	r = 0.753 *p* = 0.001	r = 0.779 *p* = 0.001	r = 0.350 *p* = 0.129

**Table 9:** BestKeeper-based evaluation of the independence of pre-established reference-gene пpanels from target-gene expression in 3T3-L1 cells.

Top Ranked Reference Gane/Pair	Gene 1 vs. BK Index, r	Gene 2 vs. BK Index, r	Target 1 *Fabp4*, r (*p*)	Target 2 *Fasn*, r (*p*)	Target 3 *Itgb1*, r (*p*)	Target 4 *Ccn1*, r (*p*)
*Nono* + *Ywhaz*	r = 0.951 *p* = 0.001	r = 0.947 *p* = 0.001	r = −0.586 *p* = 0.007	r = −0.544 *p* = 0.013	r = 0.865 *p* = 0.001	r = 0.708 *p* = 0.001
*Ywhaz* + *Rplp0*	r = 0.975 0.001	r = 0.987 *p* = 0.001	r = −0.490 *p* = 0.029	r = −0.553 *p* = 0.011	r = 0.695 *p* = 0.001	r = 0.815 *p* = 0.001
*Hprt* + *Ywhaz*	r = 0.701 *p* = 0.001	r = 0.769 *p* = 0.001	r = 0.185 ns	r = 0.092 ns	r = 0.234 ns	r = 0.488 *p* = 0.029
*Hprt* + *Nono*	r = 0.516 *p* = 0.020	r = 0.674 *p* = 0.001	r = −0.055 ns	r = −0.033 ns	r = 0.483 *p* = 0.031	r = 0.465 *p* = 0.039

**Table 10:** BestKeeper-based assessment of target-gene independence after addition of Tbp to pre-established reference-gene pairs in 3T3-L1 cells.

Top Ranked Reference Gane/Pair	Gene 1 vs. BK Index, r	Gene 2 vs. BK Index, r	Target 1 *Fabp4*, r (*p*)	Target 2 *Fasn*, r (*p*)	Target 3 *Itgb1*, r (*p*)	Target 4 *Ccn1*, r (*p*)
*Nono* + *Ywhaz* +*Tbp*	r = 0.812 *p* ≤ 0.001	r = 0.900 *p* ≤ 0.01	r = −0.441 *p* = 0.05	r = −0.387 ns	r = 0.659 *p* ≤ 0.01	r = 0.526 *p* ≤ 0.01
	Tbp: r = 0.461, *p* ≤ 0.05					
*Ywhaz* + *Rplp0* + *Tbp*	r = 0.975 *p* ≤ 0.001	r = 0.987 *p* ≤ 0.001	r = −0.374 ns	r = −0.416 ns	r = 0.533 *p* ≤ 0.01	r = 0.645 *p* ≤ 0.01
	Tbp: r = 0.446, *p* ≤ 0.05					
*Hprt* + *Ywhaz* + *Tbp*	r = 0.734 *p* ≤ 0.001	r = 0.603 *p* ≤ 0.01	r = 0.213 ns	r = 0.166 ns	r = 0.047 ns	r = 0.231 ns
	Tbp: r = 0.773, *p* ≤ 0.001					
*Hprt* + *Nono* + *Tbp*	r = 0.629 *p* ≤ 0.001	r = 0.414 ns	r = 0.063 ns	r = 0.099 ns	r = 0.188 ns	r = 0.182 ns
	Tbp: r = 0.772, *p* ≤ 0.001					

## Data Availability

The original contributions presented in this study are included in the article. Further inquiries can be directed to the author.

## Supplementary Materials

The following supporting information can be downloaded at: https://www.mdpi.com/article/10.3390/life16101637/s1, Table S1: Gene-specific amplification efficiencies for RT-qPCR assays in AML12 and 3T3-L1 cells; Table S2: Raw Cq values of candidate reference and target genes in 3T3-L1 cells cultured under different 2D and 3D conditions; Table S3: Raw Cq values of candidate reference and target genes in AML12 cells cultured under different 2D and 3D conditions; Table S4: Principal component analysis of candidate reference-gene Cq profiles in AML12 and 3T3-L1 cells; Figure S1: Comprehensive stability ranking of candidate reference genes generated by RefFinder in AML12 and 3T3-L1 cells; Figure S2: Stability ranking of candidate reference genes using the comparative ΔCq method in AML12 and 3T3-L1 cells; Figure S3: Sensitivity of target-gene expression results to alternative normalization strategies in AML12 cells; Figure S4: Sensitivity of target-gene expression results to alternative normalization strategies in 3T3-L1 cells.

## Abbreviations

The following abbreviations are used in this manuscript:

*18S*	18S ribosomal RNA
3T3-L1	Mouse embryonic fibroblast cell line used as an adipogenesis model
*Actb*	β-actin
AML12	Alpha mouse liver 12
*B2m*	Beta-2 microglobulin
BK	BestKeeper Index
*Ccn1*	Cellular communication network factor 1
cDNA	Complementary deoxyribonucleic acid
Ct	Cycle threshold
ΔCt	delta Ct
CV	Coefficient of variation
DMEM	Dulbecco’s Modified Eagle’s Medium
DMSO	Dimethyl sulfoxide
*Fabp4*	Fatty acid-binding protein 4
*Fasn*	Fatty acid synthase
*Gapdh*	Glyceraldehyde-3-phosphate dehydrogenase
HKGs	Housekeeping genes
*Hmbs*	Hydroxymethylbilane synthase
*Hprt*	Hypoxanthine guanine phosphoribosyl transferase
IBMX	3-Isobutyl-1-methylxanthine
IC	Differentiated adipocytes (positive control)
*Itgb1*	integrin beta 1 (fibronectin receptor beta)
LMM	Linear mixed model
MM	Adipocyte maintenance medium
*Nono*	Non-POU domain-containing octamer-binding protein
PBS	Phosphate-buffered saline
PCA	Principal component analysis
*Ppia*	Peptidylprolyl isomerase A
RNA	Ribonucleic acid
*Rplp0*	Ribosomal protein, large, P0
RT-qPCR	Reverse transcription quantitative polymerase chain reaction
SD	Standard deviation
SEM	Standard error of the mean
*Tbp*	TATA-box binding protein
*Ywhaz*	Tyrosine 3-monooxygenase/tryptophan 5-monooxygenase activation protein, zeta polypeptide
