# Supplementary material for: Strategies for Selecting Reliable Reference-Gene Combinations Across 2D and 3D Cell Culture Models
Source: Life (Basel). 2026 Sep 30;16(10):1637. doi: 10.3390/life16101637 (PMC13706404; doi:10.3390/life16101637)
Supplement: Supplementary file 1 [file life-16-01637-s001.zip › life-4571882-supplementary.pdf]

life

MDPI

Supplementary Table S1. Gene-specific amplification efficiencies for RT-qPCR assays in AML12 and 3T3-L1 cells

Gene	AML12 E (%)	3T3-L1 E (%)	Gene	AML12 E (%)	3T3-L1 E (%)	Gene	AML12 E (%)	3T3-L1 E (%)
18S	101.2	101.5	*Nono*	95	98.8	*Alb*	98.8	-
Actb	101.1	106.6	*Ppia*	101	102	*Hnf4a*	92.2	-
B2m	102	100.1	*Rplp0*	102.1	98.1	*Fabp4*	-	102
Gaph	103.4	100.2	*Tbp*	91.2	96.5	*Fasn*	-	98.9
Hmbs	100.8	105.6	*Ywhaz*	100.1	101.7	*Itgb1*	92.6	90.6
Hprt	100.7	99.5				*Ccn1*	102	102

Supplementary Table S2. Raw Cq values of candidate reference and target genes in 3T3-L1 cells cultured under different 2D and 3D conditions

Sample	Culture condition	18S	*Actb*	*B2m*	*Gapdh*	*Hmbs*	*Hprt*	*Nono*	*Ppia*	*Rplp0*	*Tbp*	*Ywhaz*	*Fabp4*	*Fasn*	*Itgb1*	*Ccn1*
1	2D Coll	12.25	18.76	21.25	20.19	28.96	26.48	24.40	21.36	21.05	28.35	23.39	29.23	27.41	24.81	28.36
2	2D Coll	11.60	18.00	20.01	19.12	27.40	25.87	23.75	20.62	20.00	27.96	22.32	28.48	26.35	23.50	27.41
3	2D Coll	11.85	18.22	20.46	19.41	27.57	25.93	24.21	21.05	20.35	28.27	22.50	24.71	26.24	23.81	27.37
4	2D Coll	11.51	17.77	20.19	19.10	27.17	25.55	23.41	20.40	19.91	27.78	22.08	27.20	26.09	23.31	27.01
5	2D Coll	11.49	17.81	20.16	19.05	27.44	25.91	23.58	20.43	19.90	27.91	22.16	25.93	25.83	23.50	27.47
6	3D Coll	15.28	20.65	21.52	17.24	26.57	25.28	24.95	21.55	20.94	27.65	23.03	19.82	23.98	25.46	28.82
7	3D Coll	15.78	21.04	21.46	17.43	26.74	25.20	24.85	21.85	21.01	27.59	23.16	19.66	24.48	25.63	28.86
8	3D Coll	15.02	20.62	21.18	17.03	26.32	24.83	24.34	21.23	20.53	27.39	22.69	18.33	23.76	24.99	28.48
9	3D Coll	17.72	21.08	21.27	17.58	26.37	24.98	24.84	21.26	20.69	27.70	22.90	18.74	23.84	25.45	27.93
10	3D Coll	15.67	21.08	21.92	17.76	26.57	25.15	24.65	21.60	20.77	27.62	22.67	19.37	24.38	25.25	28.69
11	Sph Coll	12.15	17.99	21.21	18.62	27.65	25.77	24.51	20.41	20.45	28.50	22.59	22.15	25.44	24.68	26.77
12	Sph Coll	11.97	17.88	21.49	18.74	27.90	25.63	24.49	20.59	20.44	28.55	22.69	22.16	25.29	24.47	26.94
13	Sph Coll	11.96	17.68	20.40	18.83	27.55	25.34	24.01	20.22	19.96	28.86	22.49	22.60	25.50	24.21	26.43
14	Sph Coll	12.03	17.83	20.64	19.06	27.63	25.59	24.14	20.55	20.28	28.57	22.60	22.91	25.65	24.19	26.43
15	Sph Coll	12.06	17.64	20.38	18.33	27.05	25.16	23.87	20.10	20.19	27.85	22.17	22.30	25.23	23.80	26.38
16	TC	12.19	18.68	21.72	18.26	27.24	25.43	24.71	21.24	21.07	28.39	23.20	20.44	23.87	24.73	30.13
17	TC	12.07	18.63	21.76	18.28	27.29	25.81	24.46	21.47	21.33	28.84	23.11	20.07	24.06	24.62	28.88
18	TC	11.75	18.58	21.70	18.27	27.44	25.42	24.31	21.43	21.11	28.09	23.05	20.28	23.47	24.43	29.23
19	TC	12.00	18.60	21.65	18.19	27.15	25.64	24.45	21.40	21.18	28.59	23.03	20.35	23.63	24.39	28.69
20	TC	11.79	18.49	21.65	18.06	27.14	25.69	24.57	21.23	21.14	27.86	23.05	20.21	23.46	24.41	29.91

Supplementary Table S3. Raw Cq values of candidate reference and target genes in AML12 cells cultured under different 2D and 3D condition

Sample	Culture condition	18S	Actb	B2M	Gapdh	Hmbs	Hprt	Nono	Ppia	Rplp0	Tbp	Ywhaz	Alb	Hnf4a	Itgb1	Ccn1
1	2D Coll	12.95	17.68	22.96	18.58	26.26	22.31	24.16	20.03	19.68	27.88	21.77	25.37	25.62	24.96	24.85
2	2D Coll	12.81	17.73	22.59	18.44	26.24	22.24	22.95	20.12	19.65	28.12	21.61	26.16	26.01	24.72	25.16
3	2D Coll	13.31	18.19	22.86	18.56	26.52	22.44	23.68	20.16	20.01	27.82	21.91	26.81	26.34	25.13	25.07
4	2D Coll	13.07	18.19	22.69	18.58	26.73	22.46	24.04	20.27	20.08	28.13	21.84	27.06	26.03	24.62	25.09
5	2D Coll	12.95	17.69	22.80	18.30	26.53	22.28	24.15	20.11	19.70	27.96	21.80	26.43	25.98	24.64	24.90
6	3D Coll	12.64	17.26	22.08	17.40	25.91	21.46	23.71	19.88	19.07	27.19	21.15	24.56	25.08	24.13	24.08
7	3D Coll	13.19	17.66	22.03	17.49	25.05	21.61	23.55	19.57	19.19	27.20	21.36	24.20	25.38	24.15	24.35
8	3D Coll	12.62	16.99	21.93	17.18	24.21	21.36	23.04	19.08	18.93	27.68	20.58	23.98	24.62	23.62	24.56
9	3D Coll	13.25	17.79	22.12	17.54	25.92	21.67	23.49	19.63	19.62	27.85	21.18	24.20	25.29	24.45	24.89
10	3D Coll	12.75	17.22	21.95	17.34	25.56	21.47	23.04	19.34	19.22	27.12	20.89	24.18	24.88	23.93	24.16
11	Sph Coll	13.80	18.21	22.34	17.86	26.25	22.34	23.64	20.21	19.73	28.02	21.83	26.76	26.49	25.26	26.32
12	Sph Coll	13.04	17.78	22.17	17.29	26.13	22.14	23.56	20.15	19.35	27.79	21.55	25.91	25.82	24.22	25.89
13	Sph Coll	13.29	17.67	21.62	16.88	25.82	21.91	23.08	19.61	19.02	27.39	21.18	26.04	25.41	23.99	25.20
14	Sph Coll	12.71	17.55	22.36	17.16	26.28	22.22	23.51	20.04	19.47	28.19	21.27	26.76	25.59	24.21	25.98
15	Sph Coll	12.87	17.63	21.81	17.26	26.09	22.14	23.11	19.74	19.19	27.36	21.22	26.20	25.59	24.18	25.46
16	TC	13.80	18.34	22.16	17.59	26.11	21.67	23.83	20.19	19.50	27.46	21.81	29.11	27.11	24.84	24.65
17	TC	13.27	18.40	22.53	17.76	26.47	22.11	23.73	20.54	19.68	27.50	21.90	29.92	26.61	24.36	25.14
18	TC	13.11	18.22	22.13	17.40	26.30	21.69	23.50	20.27	19.45	27.45	21.57	28.90	26.56	24.21	24.99
19	TC	12.32	17.67	22.42	17.19	26.23	21.74	23.23	20.42	19.58	27.43	21.30	28.87	26.28	23.58	24.33
20	TC	13.10	18.12	22.23	17.56	26.30	21.78	23.50	20.42	19.42	27.40	21.50	28.78	26.38	24.01	24.76

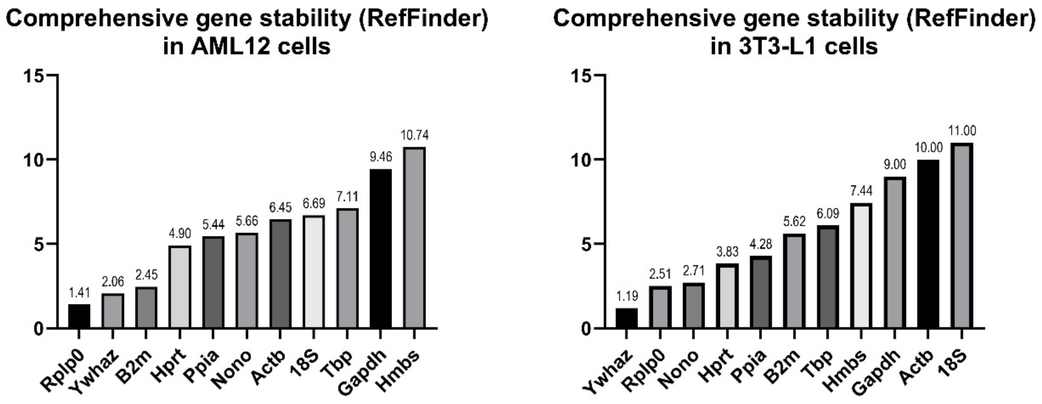

(**a**) (**b**)

Supplementary Figure S1. Comprehensive stability ranking of candidate reference genes generated by RefFinder in (a) AML12 and (b) 3T3-L1 cells. RefFinder integrates rankings derived from commonly used reference-gene stability approaches into a comprehensive ranking, with lower values indicating greater expression stability. The analysis was included as a supplementary robustness assessment. Because RefFinder operates on Cq-based input and does not explicitly account for gene-specific PCR amplification efficiencies, its results were considered supportive.

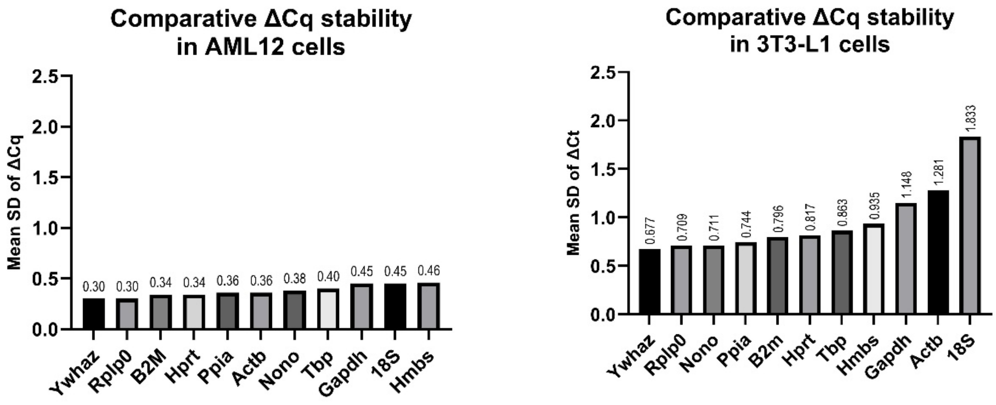

(**a**) (**b**)

Supplementary Figure S2. Stability ranking of candidate reference genes using the comparative ΔCq method in (a) AML12 and (b) 3T3-L1 cells. Gene stability was assessed from the mean standard deviation (SD) of pairwise ΔCq values across samples. Lower mean SD values indicate greater expression stability. The comparative ΔCq analysis was included as a supplementary robustness assessment and was based on Cq values without explicit correction for gene-specific PCR amplification efficiency.

life

MDPI

Supplementary Table S4. Principal component analysis of candidate reference-gene Cq profiles in AML12 and 3T3-L1 cells

A. Variance explained by the first two principal components

Gene	AML12 PC1	AML12 PC2	3T3-L1 PC1	3T3-L1 PC2
18S	0.420	−0.682	0.819	−0.364
Actb	0.762	−0.595	0.915	−0.207
B2m	0.840	0.434	0.778	0.509
Gapdh	0.794	0.359	−0.842	0.386
Hmbs	0.837	−0.115	−0.653	0.671
Hprt	0.818	0.242	−0.588	0.667
Nono	0.705	−0.028	0.849	0.362
Ppia	0.788	−0.266	0.837	0.369
Rplp0	0.927	0.137	0.708	0.650
Tbp	0.616	0.513	−0.462	0.643
Ywhaz	0.948	−0.238	0.653	0.717

B. Loadings of candidate reference genes on PC1 and PC2

Sample	Culture condition	AML12 PC1	AML12 PC2	3T3-L1 PC1	3T3-L1 PC2
1	2D Coll	2.644	1.679	−1.257	4.055
2	3D Coll	−2.492	0.178	3.568	−0.560
3	Sph Coll	2.446	−1.059	−1.327	0.630
4	2D TC	1.000	−2.602	1.210	1.569
5	2D Coll	1.279	1.747	−2.895	−1.150
6	3D Coll	−2.304	−0.765	3.902	−0.383
7	Sph Coll	0.075	−0.205	−1.186	1.046
8	2D TC	2.366	−1.405	0.781	2.464
9	2D Coll	3.575	0.392	−1.948	0.370
10	3D Coll	−5.542	1.795	2.752	−2.663
11	Sph Coll	−2.824	−1.325	−2.675	−0.631
12	2D TC	0.108	−1.529	0.852	1.245
13	2D Coll	4.050	0.919	−3.141	−2.228
14	3D Coll	−0.748	−0.016	3.596	−1.777
15	Sph Coll	0.022	1.408	−2.082	0.141
16	2D TC	−0.729	0.581	0.816	1.702
17	2D Coll	2.640	1.354	−3.316	−1.452
18	3D Coll	−3.879	0.409	3.272	−1.110
19	Sph Coll	−1.954	−0.274	−1.983	−2.390
20	2D TC	0.268	−1.283	1.059	1.124

C. Sample scores on PC1 and PC2

Cell line	Principal component	Eigenvalue	Proportion of variance (%)	Cumulative variance (%)
AML12	PC1	6.716	61.06	61.06
AML12	PC2	1.619	14.71	75.77
3T3-L1	PC1	6.156	55.96	55.96
3T3-L1	PC2	3.094	28.13	84.09

Note: PCA was performed on the Cq profiles of 11 candidate reference genes across 20 samples per cell line. The first two principal components were retained according to the Kaiser criterion (eigenvalue > 1). Loadings indicate the direction and strength of the contribution of each candidate reference gene to the respective component, whereas scores represent the position of individual samples in the PCA space. PC, principal component; TC, tissue-culture plastic; Coll, collagen; Sph, spheroid.

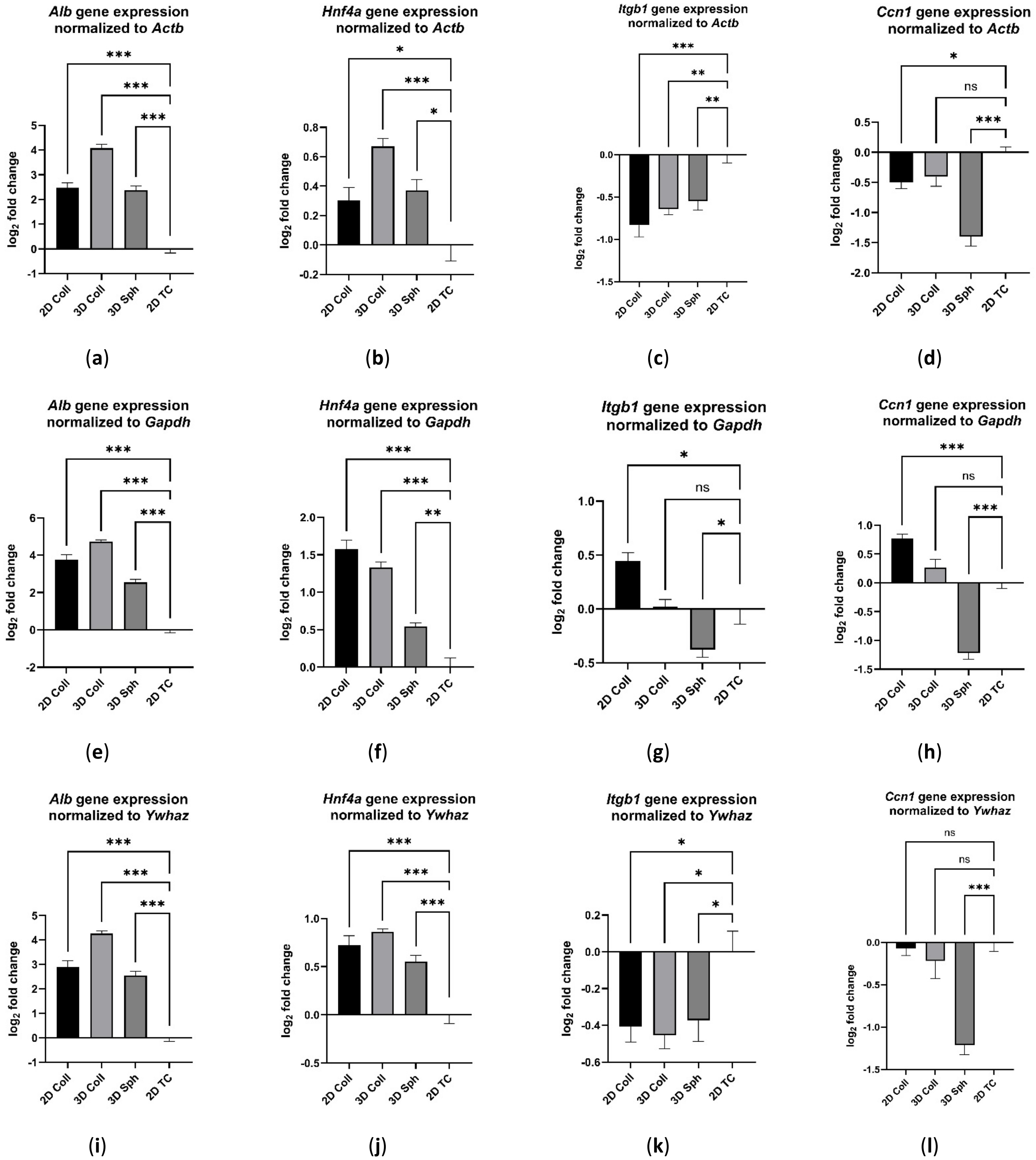

Alb gene expression normalized to *Actb*
Hnf4a gene expression normalized to *Actb*
Itgb1 gene expression normalized to *Actb*
Ccn1 gene expression normalized to *Actb*
Alb gene expression normalized to *Gapdh*
Hnf4a gene expression normalized to *Gapdh*
Itgb1 gene expression normalized to *Gapdh*
Ccn1 gene expression normalized to *Gapdh*
Alb gene expression normalized to *Ywhaz*
Hnf4a gene expression normalized to *Ywhaz*
Itgb1 gene expression normalized to *Ywhaz*
Ccn1 gene expression normalized to *Ywhaz*
$\log_2$ fold change
2D Coll
3D Coll
3D Sph
2D TC
***
**
*
ns
(a)
(b)
(c)
(d)
(e)
(f)
(g)
(h)
(i)
(j)
(k)
(l)

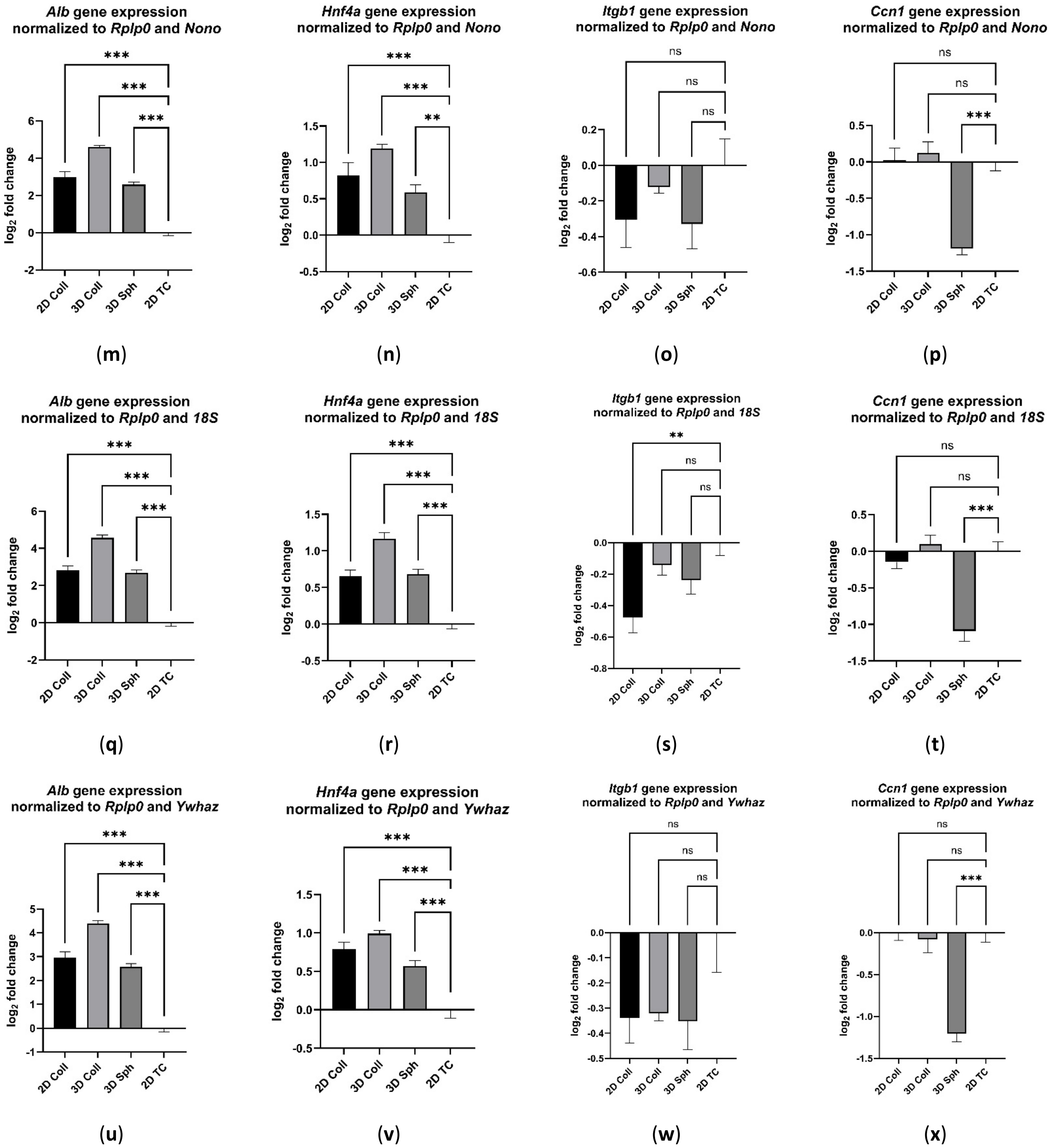

Supplementary Figure S3. Sensitivity of target-gene expression results to alternative normalization strategies in AML12 cells. Relative expression of *Alb*, *Hnf4a*, *Itgb1*, and *Ccn1* was evaluated across 2D collagen (2D Coll), 3D collagen (3D Coll), collagen spheroid (Sph Coll), and tissue culture control (TC) conditions using alternative normalization strategies: (a–d) *Actb* alone, (e–h) *Gapdh* alone, (i–l) *Ywaz* alone, (m–p) *Rplp0* + *Nono*, (q–t) *Rplp0* + *18S*, and (u–x) *Rplp0* + *Ywhaz*. Efficiency-corrected relative

quantities of the target genes were normalized either to the corresponding single reference gene or to the geometric mean of the indicated reference-gene pair. Values are presented as $\log_2$ (fold change) relative to TC; the horizontal line at 0 indicates no change relative to the calibrator. Data are presented as mean ± SD (n = 5 biological replicates). Statistical comparisons were performed separately for each target gene using ordinary one-way ANOVA followed by Dunnett's multiple-comparisons test versus TC on $\log_2$-transformed values. $^{*}p < 0.05$, $^{**}p < 0.01$, $^{***}p < 0.001$, $^{****}p < 0.0001$; ns, not significant.

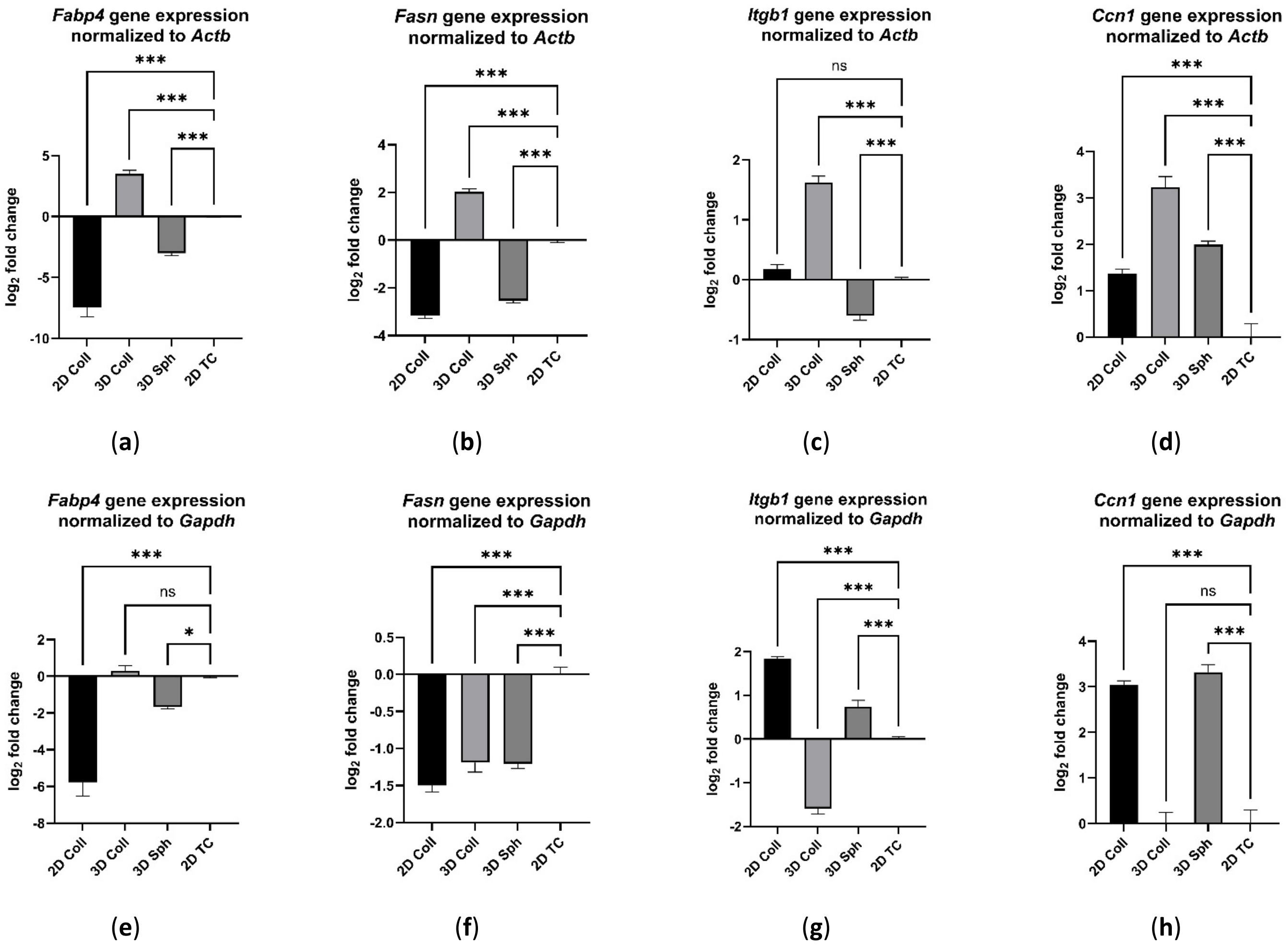

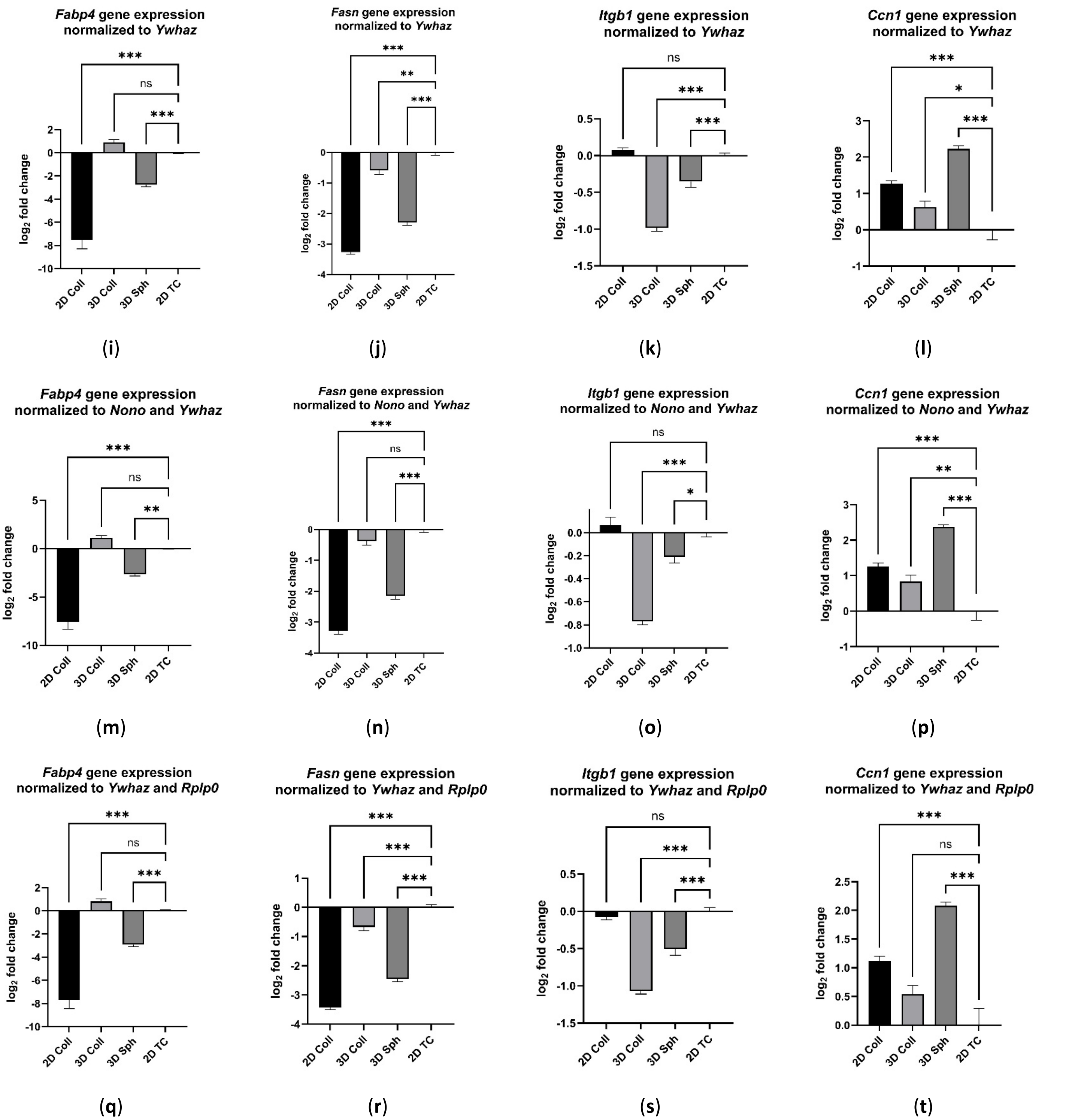

Supplementary Figure S4. Sensitivity of target-gene expression results to alternative normalization strategies in 3T3-L1 cells. Relative expression of *Alb*, *Hnf4a*, *Itgb1*, and *Ccn1* was evaluated across 2D collagen (2D Coll), 3D collagen (3D Coll), collagen spheroid (Sph Coll), and tissue culture control (TC) conditions using alternative normalization strategies: (a–d) *Actb* alone, (e–h) *Gapdh* alone, (i–l) *Ywhaz* alone, (m–p) *Nono* + *Ywhaz*, and (q–t) *Ywhaz* + *Rplp0*. Efficiency-corrected relative quantities of the target genes were normalized either to the corresponding single reference gene or to the geometric mean of the indicated

reference-gene pair. Values are presented as $\log_2$ (fold change) relative to TC; the horizontal line at 0 indicates no change relative to the calibrator. Data are presented as mean ± SD (n = 5 biological replicates). Statistical comparisons were performed separately for each target gene using ordinary one-way ANOVA followed by Dunnett's multiple-comparisons test versus TC on $\log_2$-transformed values. *$p < 0.05$, **$p < 0.01$, ***$p < 0.001$, ****$p < 0.0001$; ns, not significant.
